# ROCK1 induces dopaminergic nerve cell apoptosis via the activation of Drp1-mediated aberrant mitochondrial fission in Parkinson’s disease

**DOI:** 10.1038/s12276-019-0318-z

**Published:** 2019-10-02

**Authors:** Qian Zhang, Changpeng Hu, Jingbin Huang, Wuyi Liu, Wenjing Lai, Faning Leng, Qin Tang, Yali Liu, Qing Wang, Min Zhou, Fangfang Sheng, Guobing Li, Rong Zhang

**Affiliations:** Department of Pharmacy, The Second Affiliated Hospital of Army Medical University, 400037 Chongqing, China

**Keywords:** Apoptosis, Parkinson's disease

## Abstract

Dopamine deficiency is mainly caused by apoptosis of dopaminergic nerve cells in the substantia nigra of the midbrain and the striatum and is an important pathologic basis of Parkinson’s disease (PD). Recent research has shown that dynamin-related protein 1 (Drp1)-mediated aberrant mitochondrial fission plays a crucial role in dopaminergic nerve cell apoptosis. However, the upstream regulatory mechanism remains unclear. Our study showed that Drp1 knockdown inhibited aberrant mitochondrial fission and apoptosis. Importantly, we found that ROCK1 was activated in an MPP^+^-induced PD cell model and that ROCK1 knockdown and the specific ROCK1 activation inhibitor Y-27632 blocked Drp1-mediated aberrant mitochondrial fission and apoptosis of dopaminergic nerve cells by suppressing Drp1 dephosphorylation/activation. Our in vivo study confirmed that Y-27632 significantly improved symptoms in a PD mouse model by inhibiting Drp1-mediated aberrant mitochondrial fission and apoptosis. Collectively, our findings suggest an important molecular mechanism of PD pathogenesis involving ROCK1-regulated dopaminergic nerve cell apoptosis via the activation of Drp1-induced aberrant mitochondrial fission.

## Parkinson’s disease: Looking back identifies a new target

Researchers in China have revealed how a protein molecule plays an early part in the molecular steps that can lead to Parkinson’s disease, which is caused by the death of nerve cells that make the neurotransmitter dopamine. Disruption of mitochondria, the energy-generating bodies inside cells, was already known to lead to the death of dopamine-producing cells. Rong Zhang, Guobing Li and colleagues at The Second Affiliated Hospital of Army Medical University in Chongqing, China traced the chain of cause and effect back to a protein called ROCK-1. Using a mouse model of Parkinson’s disease, they found that ROCK-1 activates another protein previously shown to trigger the disruption of mitochondria. ROCK-1’s early role in the sequence might make it a suitable target for treatment using drugs that inhibit its activity.

## Introduction

Parkinson’s disease (PD), which often occurs in elderly patients, is a neurodegenerative disease characterized by dopamine deficiency mainly caused by nigrostriatal dopaminergic nerve cell apoptosis. As the population continues to age, the incidence of PD continues to increase yearly^1^. As the pathogenesis remains obscure, therapeutic options for PD are mainly symptomatic therapies, and levodopa (L-DOPA) has been the most effective drug since the 1960s^2^. However, the long-term administration of L-DOPA has limited clinical applications due to adverse side effects associated with long-term use^3^. Therefore, the molecular mechanism of nigrostriatal dopaminergic nerve cell apoptosis needs to be elucidated and is of great significance for improving therapeutic strategies for the treatment of PD.

Studies have found a close link between mitochondrial dysfunction and PD pathogenesis^4–6^. Mitochondria participate in the regulation of cellular physiological functions, including cellular homeostasis, cell growth, division, and energy metabolism, specifically as it relates to apoptosis^7^. Mitochondrial dysfunction is critical to PD pathogenesis, and the restoration of mitochondrial function may reduce dopaminergic nerve cell apoptosis, thereby attenuating dopamine failure and improving PD symptoms^8^. Moreover, mitochondria are dynamic and undergo frequent fission and fusion under the regulation of a variety of dynamic proteins, such as dynamin-related protein 1 (Drp1), mitochondria fission protein 1 (Fis1), and mitochondrial fission factor (Mff) for fission and optic atrophy 1 (Opa1) and mitofusin (Mfn) for fusion. Recent studies have shown that Drp1-induced aberrant mitochondrial fission plays a vital role in dopaminergic nerve cell apoptosis in PD. The enhancement of Drp1 promotes mitochondrial fission and PD-associated dopaminergic nerve cell apoptosis, whereas the inhibition of Drp1 reverses aberrant mitochondrial fission, reduces nerve cell apoptosis, and improves PD symptoms^5,9–12^. Drp1 is a GTPase; once Drp1 is activated, Drp1 is translocated from the cytosol to the outer mitochondrial membrane (i.e., mitochondrial translocation), forms a ring structure around the mitochondria, and changes the distance and angle of molecules, gradually compressing the mitochondria until they are fractured by GTP hydrolysis, resulting in the fission of mitochondria followed by cytochrome c (Cyto C) release and caspase activation, eventually leading to apoptosis^13–16^. However, the upstream regulatory mechanism of Drp1-mediated mitochondrial fission in PD has not yet been explored.

Rho-associated coiled-coil protein kinase 1 (ROCK1) is a member of the Ras protein family and has a molecular weight of 160 kDa; it plays an important regulatory role in cancer cell growth and survival, as well as the invasion and metastasis of neoplasm^17^. In the field of cancer research, ROCK1 has been reported to be cleaved into activated ROCK1, which has a molecular weight of 130 kDa, through the proteolytic cleavage of its C-terminal autoinhibitory domain eventually leading to apoptosis^18^. Importantly, activated ROCK1 has been found to play a crucial role in regulating mitochondrial fission via the activation of Drp1 in human breast cancer cells^19^. In addition, other reports have demonstrated that, in the central nervous system, the specific ROCK1 activation inhibitor Y-27632 decreases dopaminergic nerve cell death in mice and primary neuron/glia cultures^20,21^, but the mechanisms remain elusive. Based on the above, we propose that ROCK1 may be involved in the pathogenesis of PD as an important upstream regulator of Drp1.

In this study, we confirmed that Drp1-mediated aberrant mitochondrial fission participates in the pathogenesis of PD. Furthermore, we evaluated the regulatory role of ROCK1 in dopaminergic nerve cell apoptosis in PD. We found that ROCK1 is activated in PD and that ROCK1 knockdown or pretreatment with the ROCK1 activation inhibitor Y-27632 inhibits Drp1-mediated aberrant mitochondrial fission and dopaminergic nerve cell apoptosis in vitro and in vivo and significantly improves PD symptoms in a mouse model. Our mechanistic studies revealed that activated ROCK1 promotes aberrant mitochondrial fission by inducing the dephosphorylation/activation of Drp1, resulting in dopaminergic nerve cell apoptosis and eventually leading to PD. Furthermore, we identified Y-27632 has a therapeutic effect in PD mice by suppressing Drp1-mediated aberrant mitochondrial fission and apoptosis. Our study provides a novel insight into the role of dopaminergic nerve cell apoptosis in PD and a mechanistic basis for promoting use of ROCK1 activation inhibitors for the treatment of PD.

## Materials and methods

### Reagents

1-Methyl-4-phenyl-1, 2, 3, 6-tetrahydropyridine hydrochloride (MPTP-HCl, M0896) and 1-methyl-4-phenylpyridinium iodide (MPP^+^I^−^, D048) were obtained from Sigma-Aldrich Co. (St. Louis, MO, USA). Y-27632 (sc-216067) was purchased from Santa Cruz Biotechnology (Santa Cruz, CA, USA).

### Cell lines and cell culture

American Type Culture Collection (ATCC, Manassas, VA, USA) provided PC12 cells. RPMI-1640 medium supplemented with 10% (v/v) fetal bovine serum (Gibco, 10100) was used to culture cells at 37 °C in 5% CO_2_ and 95% air.

### Plasmid constructs and lentiviral gene transfer

Drp1 short hairpin RNA (shRNA) (target sequence: 5′-CCGGGCTACTTTACTCCAACTTATTCTCGAGAATAAGTTGGAGTAAAGTAGCTTTTT-3′) and ROCK1 shRNA (target sequence: 5′-CCGGCGGTTAGAACAAGAGGTAAATCTCGAGATTTACCTCTTGTTCTAACCGTTTTT-3′) were subcloned into the pLKO.1 plasmid to construct shDrp1 and shROCK1 plasmids, respectively. A control shRNA plasmid (pLKO.1-puro plasmid, sc-108060) was purchased from Santa Cruz. The following lentiviral packaging vectors were used: pLP1, pLP2, and VSVG (Invitrogen, K4975). Following the manufacturer’s instructions, Lipofectamine 3000 (Invitrogen, L3000015) was used to cotransfect 293FT cells with the shDrp1 or shROCK1 plasmid and the abovementioned packaging vectors. After 48 h, the lentiviral supernatant was harvested and transfected into PC12 cells. PC12 cells with stable knockdown of Drp1 or ROCK1 were then selected using puromycin (5 μg/ml, Sigma, P9620).

### Dopamine detection

Cell culture supernatants were treated with 1-methyl-4-phenylpyridinium ion (MPP^+^) and carefully collected after centrifuging at 3000 rpm for 20 min. The dopamine concentration was quantified using enzyme-linked immunosorbent assays (ELISAs; Wuhan Colorful Gene Biological Technology, Wuhan, China) according to the manufacturer’s instructions.

### MTT (3-[4,5-dimethylthiazol-2-yl]−2,5-diphenyltetrazolium bromide) assay

An MTT assay was performed to determine the effects of MPP^+^ on PC12 cell viability. MTT solutions (5 mg/ml MTT, Sigma-Aldrich, Saint Louis, MO, USA) were added to the MPP^+^-treated and untreated cells. After 4 h, the absorption was read by a microplate reader (Thermo, Varioskan Flash) at 570 nm. Cell viability was normalized to that of the control group (100%).

### Mitochondrial membrane potential determination by JC-1 and rhodamine 123 staining

The JC-1 Kit (Beyotime Company, C2006) was used to measure the mitochondrial membrane potential. Briefly, the cells were seeded in 24-well plates. The MPP^+^-treated cells were incubated with the JC-1 probe for 15 min at 37 °C and washed twice in ice-cold 1 × assay buffer. CCCP (protonophore, carbonyl cyanide m-chlorophenylhydrazone, 10 µM) was used as a positive control. Fluorescence was observed by fluorescence microscopy (BX63, Olympus, Japan), and the fluorescence intensity was quantified by the ImageJ software (National Institutes of Health, USA). The fluorescence ratio of JC-1 aggregates (red) to JC-1 monomers (green) was used to determine the mitochondrial membrane potential, and the ratio was normalized to that of the control group (100%).

Rhodamine 123 staining was also used to detect the mitochondrial membrane potential. Briefly, following MPP^+^ treatment, cells were harvested and stained with 1 μM rhodamine 123 in a 5% CO_2_ incubator for 30 min at 37 °C in the dark. Subsequently, ice-cold phosphate-buffered saline (PBS) was used to wash the cells twice. The fluorescence intensity was read by a microplate reader (Thermo, Varioskan Flash) at 507 nm (excitation wavelength) and 529 nm (emission wavelength). The rhodamine 123 fluorescence was normalized to that of the control group (100%).

### Adenosine triphosphate (ATP) luminescence detection

The firefly luciferase-based ATP Determination Kit (Beyotime Company, S2006) was used to measure ATP levels according to the manufacturer’s instructions. Cells treated with various concentrations of MPP^+^ were lysed and centrifuged, and ATP detection working solution was added to the supernatant. The luminescence value, which was determined with a microplate reader (Thermo, Varioskan Flash), was used as an index of the ATP level. The ATP level was normalized to that of the control group (100%).

### Determination of apoptosis by flow cytometry

Cells were digested with trypsin, centrifuged, and washed twice with PBS. Subsequently, the cells were stained with a mixture of annexin V-fluorescein isothiocyanate (FITC) and propidium iodide (PI) (BD Biosciences, 556547). After incubation for 15 min at 25 °C in the dark, the rate of apoptosis (early apoptotic cells (annexin V-FITC^+^/PI^−^) and late apoptotic cells (annexin V-FITC^+^/PI^+^)) was analyzed by flow cytometry (FACScan, Beckman MoFlo XDP).

### Western blot analysis

Cells and tissues were lysed with cell lysis buffer containing 1 mM phenylmethylsulfonyl fluoride. The Mitochondrial Isolation Kit (Beyotime Company, C3601) was used to separate and extract mitochondrial and cytosolic lysates. The protein concentration of the lysates was determined by a BCA Protein Assay Kit (Beyotime Company, P0009). Then 15–100 μg of protein from each sample was separated using sodium dodecyl sulfate–polyacrylamide gel electrophoresis and transferred to polyvinylidene difluoride membranes. Blocking was performed with 5% fat-free milk, and the membranes were then incubated overnight with corresponding primary antibodies at 4 °C. The following antibodies were used: cleaved poly ADP ribose polymerase (PARP; 94885, 1:500), cleaved caspase-3 (9661, 1:500) (Cell Signaling Technology), Drp1 (611113, 1:500; BD Biosciences), p-Drp1 (YP0841, 1:1000; ImmunoWay), ROCK1 (ab45171, 1:2000), tyrosine hydroxylase (TH; ab112, 1:1000), COX IV (ab202554, 1:2000) (Abcam), and β-actin (A1978, 1:50,000; Sigma). The membranes were then incubated with a horseradish peroxidase-conjugated goat anti-rabbit (KPL, 074–1516) or goat anti-mouse (KPL, 074–1802) secondary antibody for 2 h at 25 °C and subsequently visualized with an enhanced chemiluminescence reagent (Bio-Rad, 170–5061). Protein densitometry was calculated by the Quantity One software (Bio-Rad, Germany) and normalized to that of the control group (100%).

### Immunofluorescence

Cells were plated on coverslips and then transfected with a DsRed-Mito plasmid (Clontech Laboratories, Inc., PT3633–5) for 48 h using Lipofectamine 3000 (Invitrogen, L3000015). After fixation with 4% paraformaldehyde for 15 min, the mitochondria were viewed under a LSM780 confocal laser scanning microscope (Zeiss, Germany). The mitochondrial length was measured in at least 10 randomly selected cells using the Imaris software (Bitplane, Zurich, Switzerland).

### Animals and treatment

All animal experiments were conducted with approval from the Laboratory Animal Welfare and Ethics Committee of Army Medical University. The approval license number is SYXK 20170002.

MPTP was used to establish a PD mouse model^22,23^. Eight-week-old male C57BL/6 mice (20–25 g) were randomly divided into four groups: the control, MPTP, Y-27632, and Y-27632+MPTP groups (eight mice per group). The mice in the MPTP and Y-27632 groups were intraperitoneally (i.p.) injected with MPTP at a dose of 30 mg/kg/day and Y-27632 at a dose of 5 mg/kg/day, respectively, once a day for 5 consecutive days^24^. The mice in the Y-27632+MPTP group was injected with a dose of MPTP (30 mg/kg/day) 30 min after the injection of Y-27632 (5 mg/kg/day). The mice in the control group were injected with an equal volume of vehicle on the same schedule. On the seventh day, after the last injection of MPTP, the mice were anesthetized with chloral hydrate (0.4 ml/100 g, i.p.). Mice were transcardially perfused with saline and fixed with 4% paraformaldehyde. The brains were immersion-fixed overnight in 4% paraformaldehyde and dehydrated for 48 h in 30% sucrose solution at 4 °C. The dehydrated brain tissues were coronally sectioned encompassing the entire substantia nigra pars compacta (SNpc) of the midbrain and striatum for immunofluorescence and immunohistochemical analysis. For western blot analysis, the mice were euthanized under anesthesia with chloral hydrate, and the brain tissues were quickly removed. The SNpc of the midbrain and striatum were dissected on ice.

### Rotarod test

During the test, the mice were placed on the rotarod (IITC Life Science, Series 8). Before the test, the mice were pretrained for 3 days. The training consisted of three consecutive runs of 5 rpm for 30 s followed by an acceleration up to 40 rpm over 5 min^23^. Each trial continued until the mice were unable to remain on the rod without falling off (maximum of 120 s)^23^. The latency for the mice to fall from the rotarod was recorded and was analyzed for statistical analyses (*n* = 3).

### Statistical analysis

The data are expressed as the means plus or minus the standard deviation (means ± S.D.) of at least three independent experiments. The statistical analysis was performed by one-way analysis of variance with Dunnett’s test or Tukey’s test by the GraphPad Prism 5.0 statistical analysis software. The significance of the differences between two groups was evaluated using *t* tests. *P < 0.05, **P < 0.01, and ***P < 0.001 were regarded as statistically significant.

## Results

### MPP^+^ inhibits dopamine release in PD cells

The degeneration of nigrostriatal dopaminergic nerve cells in PD can be modeled by the administration of the neurotoxin MPP^+^ in vitro^12^. In this work, we used MPP^+^-treated pheochromocytoma PC12 cells (a dopaminergic cell model system) as a model of PD in vitro. First, we evaluated the effects of MPP^+^-induced dopamine loss in PC12 cells using ELISAs. As shown in Supplementary Fig. 1, the exposure of PC12 cells to MPP^+^ led to a significant decrease in dopamine production in a dose-dependent manner. This result confirms that the in vitro model of PD was successfully established.

### MPP^+^ induces mitochondria-dependent apoptosis in PC12 cells

**Fig. 1:**
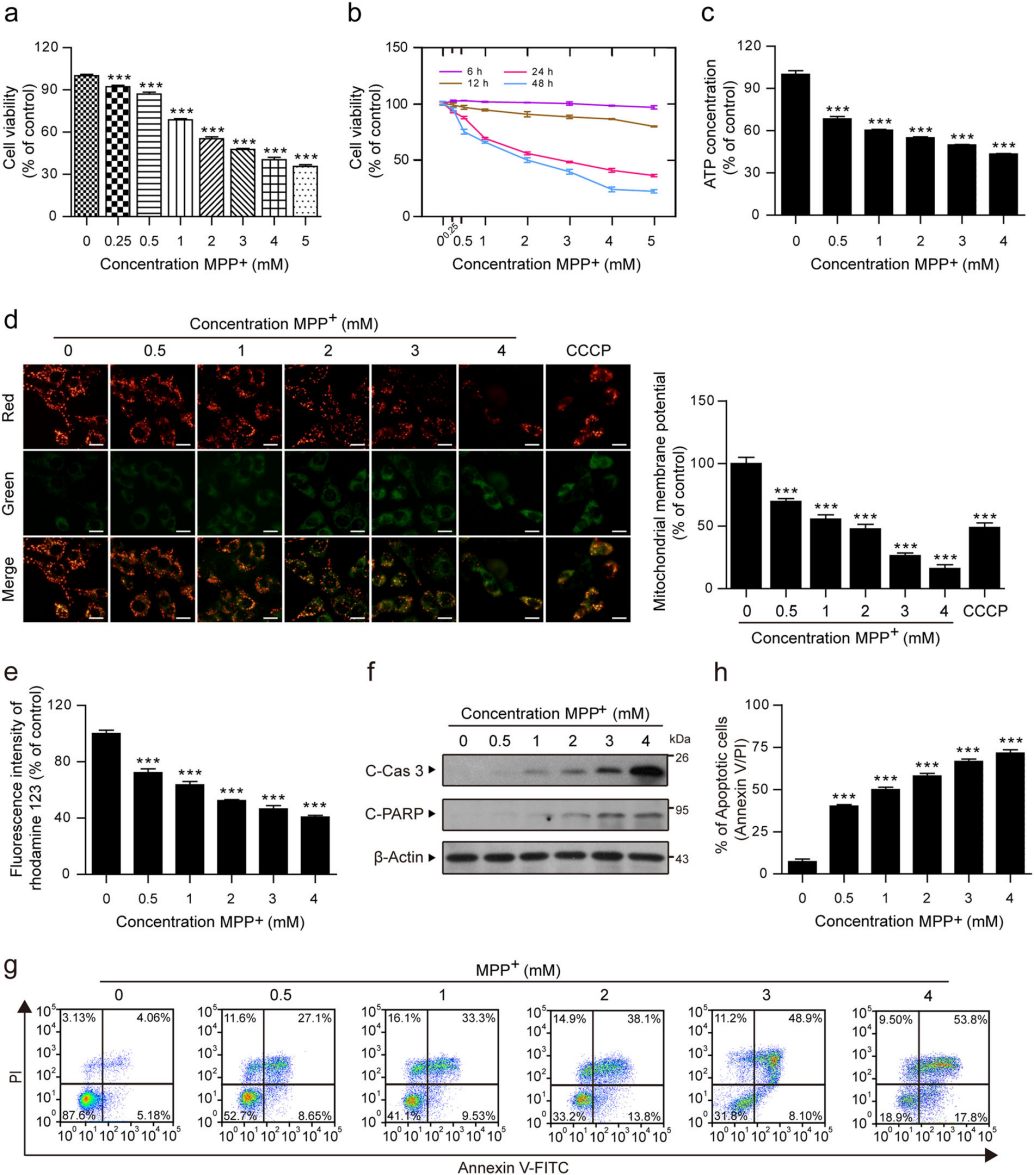
MPP^+^ induces mitochondria-dependent apoptosis in PC12 cells. PC12 cells were treated with various concentrations of MPP^+^ (0, 0.25, 0.5, 1, 2, 3, 4, and 5 mM) for 24 h (**a**) or at different time intervals (**b**), and the viability of PC12 cells was measured by the MTT assay. **c** The ATP Determination Kit was used to determine the concentration of ATP in PC12 cells treated with MPP^+^ (0, 0.5, 1, 2, 3, and 4 mM). **d** The mitochondrial membrane potential was measured by JC-1 staining. CCCP (10 µM) was used as the positive control. Scale bars: 20 μm. The fluorescence intensity ratio of red (JC-1 aggregates) to green (JC-1 monomers) fluorescence was used to represent the mitochondrial membrane potential. **e** The rhodamine 123 fluorescence intensity was detected by a microplate reader. **f** The expression of cleaved caspase-3 (C-Cas 3) and cleaved PARP (C-PARP) in whole-cell lysates was determined by western blot analysis. **g**, **h** The apoptosis rate was measured by flow cytometry using annexin V-FITC/PI staining. The data are expressed as the means ± S.D. (*n* = 3). ***P < 0.001 vs. the control group

To further explore the pathogenesis in the MPP^+^-induced PD model, we first studied the effect of MPP^+^ on cell viability as measured by the MTT assay. PC12 cells were treated with MPP^+^ at different concentrations and different time intervals. Our results showed that MPP^+^ induced significant decreases in the cell viability of PC12 cells in a dose- and time-dependent manner (Fig. 1a, b).

ATP, as the most important energy molecule, plays an important role in cellular physiological and pathogenic processes. ATP depletion is always an indicator of mitochondrial dysfunction^25–27^. As shown in Fig. 1c, the content of ATP rapidly decreased in MPP^+^-treated cells in a dose-dependent manner. The loss of the mitochondrial membrane potential is another marker of mitochondrial dysfunction^28,29^. Therefore, we examined the mitochondrial membrane potential using JC-1 and rhodamine 123 staining. The mitochondrial membrane potential of the cells was represented by the ratio of JC-1 aggregates to JC-1 monomers (i.e., the ratio of red/green fluorescence intensity). Our results showed that MPP^+^ dose-dependently decreased the red fluorescence intensity and increased the green fluorescence intensity and that the ratio of red-to-green fluorescence intensity decreased significantly (Fig. 1d). Rhodamine 123, which is specifically located in mitochondria, is also widely used to detect the mitochondrial membrane potential based on fluorescence intensity^30^. Our results showed that, in cells treated with MPP^+^, there were dose-dependent decreases in the fluorescence intensity of rhodamine 123 (Fig. 1e). Both the decrease in ATP concentration and the loss of the mitochondrial membrane potential suggest that MPP^+^ induces mitochondrial dysfunction in PC12 cells.

Mitochondrial dysfunction is an important indicator of mitochondria-dependent apoptosis^31–33^. To investigate whether MPP^+^-mediated mitochondrial dysfunction results in the induction of apoptosis, we used western blot and flow cytometry to identify apoptotic cells. We found that MPP^+^ caused the cleavage/activation of classical apoptosis-related proteins, such as caspase-3 and PARP (Fig. 1f). Consistent with these findings, MPP^+^ induced a dose-dependent increase in the percentage of apoptotic cells (Fig. 1g, h). Taken together, our findings suggest that MPP^+^ induces mitochondria-dependent apoptosis in PC12 cells.

### MPP^+^ induces Drp1-dependent aberrant mitochondrial fission in PC12 cells

**Fig. 2:**
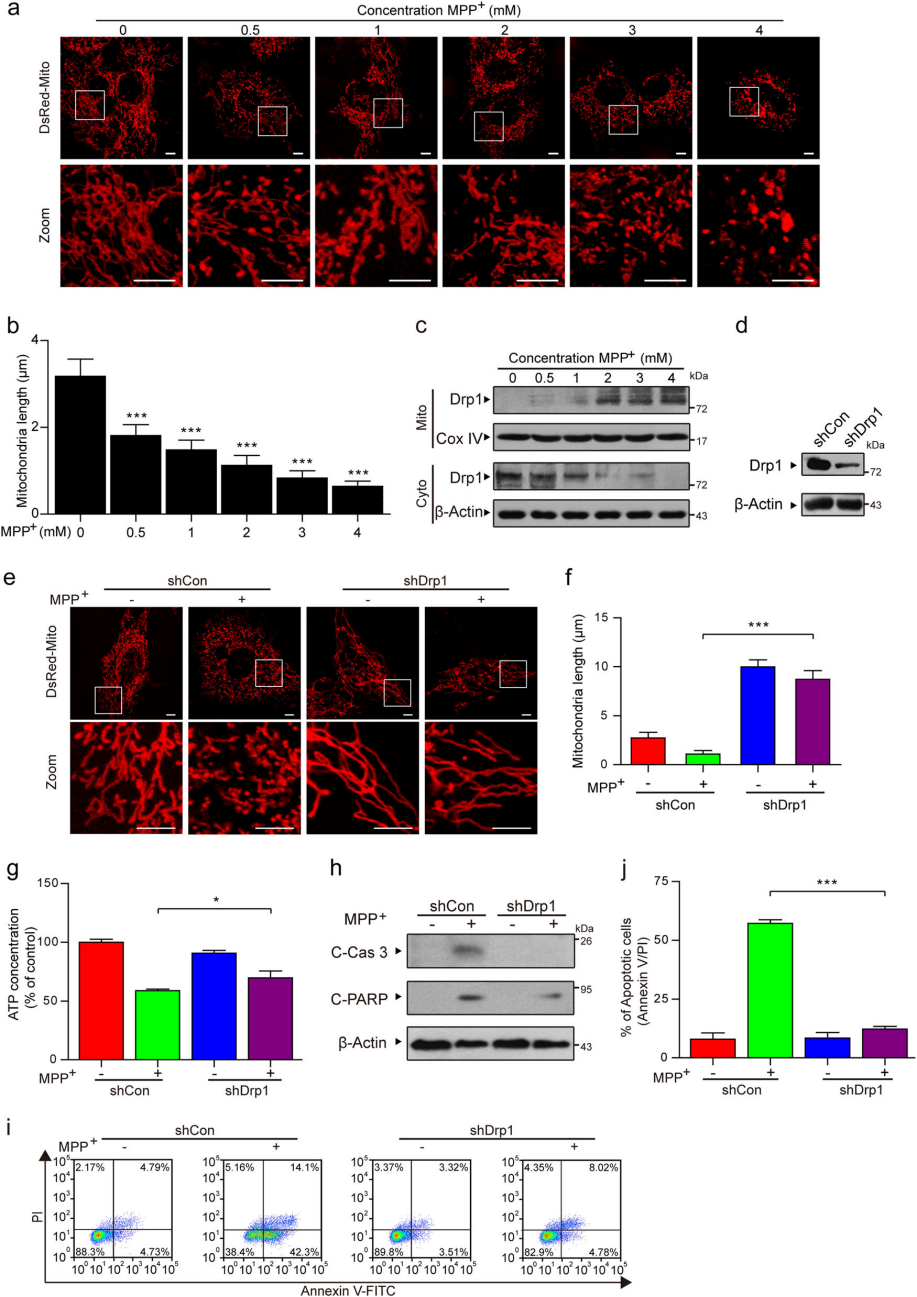
MPP^+^ induces Drp1-dependent aberrant mitochondrial fission in PC12 cells. **a** Cells were transfected with a DsRed-Mito plasmid, and the mitochondria were viewed by confocal microscopy. Scale bars: 5 μm. **b** Mitochondria length was quantified using the Imaris software. **c** PC12 cells were treated with various doses of MPP^+^ (0, 0.5, 1, 2, 3, and 4 mM) for 24 h. The expression of Drp1 in the mitochondrial lysates (Mito) and in the cytosolic fractions (Cyto) was determined by western blot analysis. **d** The stable expression of a nontargeting shRNA (shCon) or Drp1 shRNA (shDrp1) in PC12 cells was confirmed by western blot analysis. **e** Cells treated with MPP^+^ (1 mM) alone or in combination with Drp1 knockdown were transfected with a DsRed-Mito plasmid, and the mitochondria were viewed by confocal microscopy. Scale bars: 5 μm. **f** Mitochondrial length was quantified using the Imaris software. **g** The ATP Determination Kit was used to determine the concentration of ATP. **h** The expression of C-Cas 3 and C-PARP in whole-cell lysates was determined by western blot analysis. **i**, **j** The apoptosis rate was measured by flow cytometry using annexin V-FITC/PI staining. The data are expressed as the means ± S.D. (*n* = 3). *P < 0.05, ***P < 0.001

Mitochondria are dynamic organelles that frequently divide and fuse, and increasing evidence has indicated that mitochondrial fission participates in the initiation of mitochondrial apoptosis^32,33^. To examine the effects of MPP^+^ on mitochondrial fission, a DsRed-Mito plasmid was transfected into cells before MPP^+^ treatment. Confocal laser scanning microscopy indicated that the average length of the mitochondria was remarkably decreased in MPP^+^-treated cells in a dose-dependent manner (Fig. 2a, b). These results reveal that MPP^+^ induces mitochondrial apoptosis via mitochondrial fission in PC12 cells.

Drp1 is responsible for mitochondrial fission through its translocation from the cytosol to the mitochondria (i.e., mitochondrial translocation)^32,34,35^. Therefore, we next investigated whether the mitochondrial translocation of Drp1 is a key event in MPP^+^-induced mitochondrial fission. PC12 cells treated with MPP^+^ exhibited a significant increase in the levels of Drp1 in the mitochondria and a decrease in the cytosolic Drp1 levels in a dose-dependent manner (Fig. 2c). To further verify the critical function of Drp1 in MPP^+^-induced aberrant mitochondrial fission in a PD cell culture model, lentiviral shDrp1 was used to specifically suppress the expression of Drp1 (Fig. 2d). Confocal laser scanning microscopy demonstrated that Drp1 knockdown remarkably increased the average mitochondrial length, suggesting that Drp1 knockdown inhibited MPP^+^-induced aberrant mitochondrial fission (Fig. 2e, f). Compared with the transfection of shCon, the depletion of Drp1 attenuated MPP^+^-induced ATP loss (Fig. 2g). Moreover, Drp1 knockdown blocked the MPP^+^-induced activation of caspase-3 and PARP, as well as apoptosis (Fig. 2h–j). Taken together, these findings reveal that Drp1 is required for aberrant mitochondrial fission and apoptosis induced by MPP^+^.

### ROCK1 activation is involved in MPP^+^-induced aberrant mitochondrial fission and apoptosis through the dephosphorylation/activation of Drp1

**Fig. 3:**
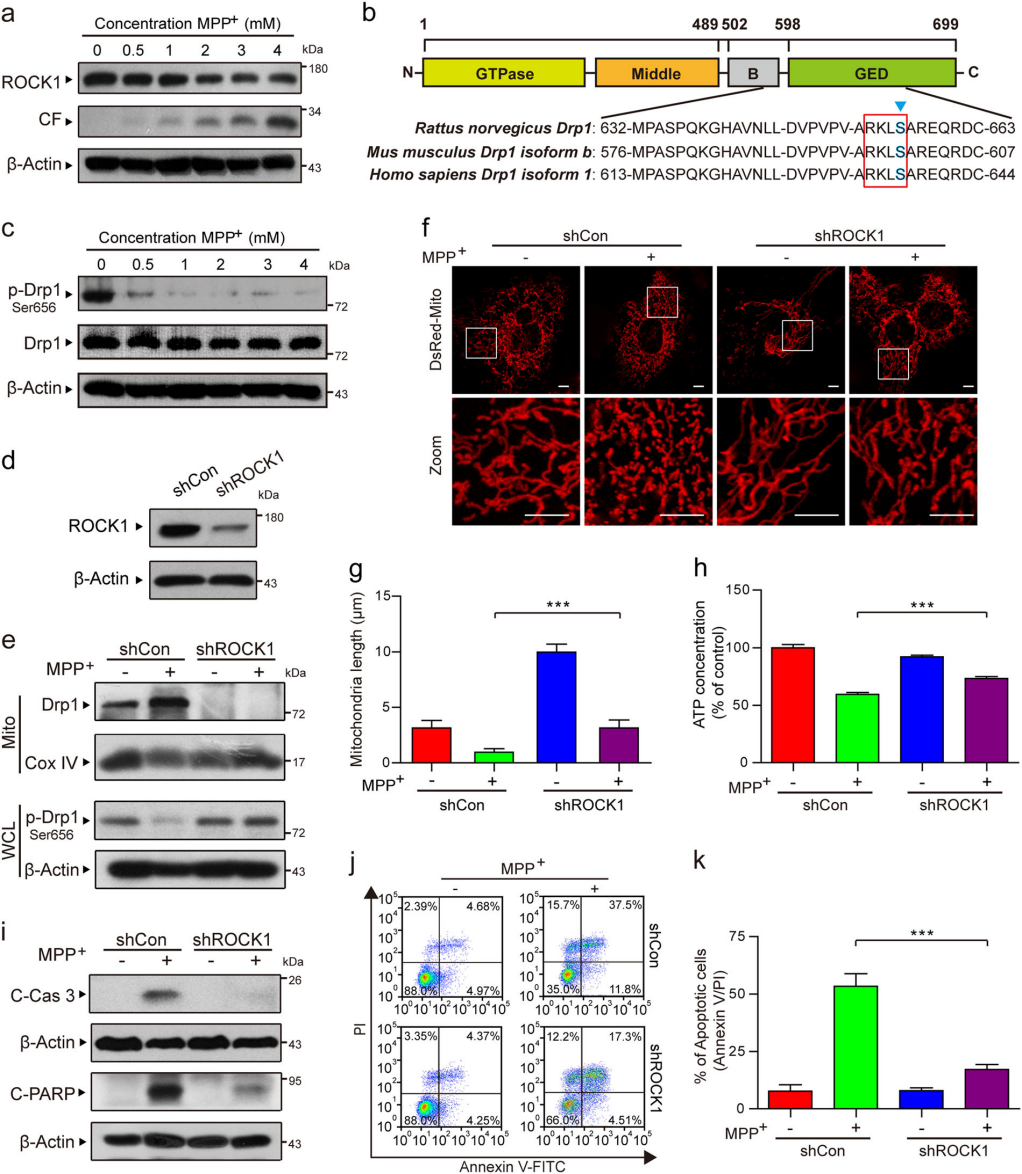
ROCK1 activation is involved in MPP^+^-induced aberrant mitochondrial fission and apoptosis through the dephosphorylation/activation of Drp1. **a** PC12 cells were treated with various concentrations of MPP^+^ (0, 0.5, 1, 2, 3, and 4 mM) for 24 h. The expression of ROCK1, CF of ROCK1, and p-Drp1 (Ser 656) in whole-cell lysates was determined by western blot analysis. CF represents the cleavage fragment of ROCK1. **b** The domain structure of Drp1. Highly conserved motifs in Drp1 isoforms were identified in Rattus, *Mus musculus* and *Homo sapiens*. **c** The expression of p-Drp1 (Ser 656) and total Drp1 in whole-cell lysates was determined by western blot analysis. **d** The stable expression of shCon or ROCK1 shRNA (shROCK1) in PC12 cells was confirmed by western blot analysis. Then the cells were treated with MPP^+^ (1 mM) alone or in combination with ROCK1 knockdown. **e** The expression of Drp1 in mitochondrial lysates (Mito) and p-Drp1 (Ser 656) in whole-cell lysates (WCL) was determined by western blot analysis. **f** Cells were transfected with a DsRed-Mito plasmid, and the mitochondria were viewed by confocal microscopy. Scale bars: 5 μm. **g** Mitochondrial length was quantified using Imaris software. **h** The ATP Determination Kit was used to determine the concentration of ATP. **i** The expression of C-Cas 3 and C-PARP in whole-cell lysates was determined by western blot analysis. **j**, **k** The apoptosis rate was measured by flow cytometry using annexin V-FITC/PI staining. The data are expressed as the means ± S.D. (*n* = 3). ***P < 0.001

It has been reported that ROCK1 plays a vital regulatory role in apoptosis^17,18^. Our results revealed that MPP^+^ dose-dependently decreased the expression of ROCK1 and increased the expression of cleaved fragment (CF) of ROCK1 (Fig. 3a). ROCK1 activation has been reported to be involved in regulating the mitochondrial translocation of Drp1 and mitochondrial fission through its dephosphorylation at Ser 637 in human breast cancer cells^19^. As shown in Fig. 3b, the GTPase effector domain of Drp1 is a serine phosphorylation site that is highly conserved among species. It has been suggested that Ser 656 in rats and Ser 600 in mice corresponds to Ser 637 in humans as ROCK substrates, which are characterized by the sequence motif R-X-X-S (where R is arginine and S is serine)^36,37^. Thus we identified that the potential phosphorylation site of rat Drp1 is Ser 656. And we next examined whether MPP^+^ phosphorylates rat Drp1 at Ser 656 in PC12 cells. A dose-dependent decrease in the phosphorylation of Drp1 at Ser 656 (i.e., an increase in the dephosphorylation of Drp1 at Ser 656) was detected in the cells exposed to MPP^+^ (Fig. 3c). In contrast, MPP^+^ treatment did not alter total Drp1 expression (Fig. 3c). To further confirm these findings, we stably knocked down ROCK1 using lentiviral shRNA (Fig. 3d). We next investigated whether ROCK1 activation is required for the MPP^+^-mediated translocation of Drp1 to the mitochondria. The knockdown of ROCK1 reversed Drp1 mitochondrial translocation and dephosphorylation at Ser 656 (Fig. 3e). ROCK1 knockdown also blocked MPP^+^-induced mitochondrial fission (Fig. 3f, g). In addition, ROCK1 knockdown inhibited MPP^+^-mediated ATP loss, caspase-3 and PARP activation, and apoptosis (Fig. 3h–k). Taken together, these results show that activated ROCK1 is involved in MPP^+^-induced aberrant mitochondrial fission and apoptosis through increased Drp1 dephosphorylation at Ser 656 in a PD cell culture model.

### The ROCK1 activation inhibitor Y-27632 attenuates MPP^+^-induced Drp1-dependent aberrant mitochondrial fission and apoptosis through the inhibition of Drp1 dephosphorylation/activation

**Fig. 4:**
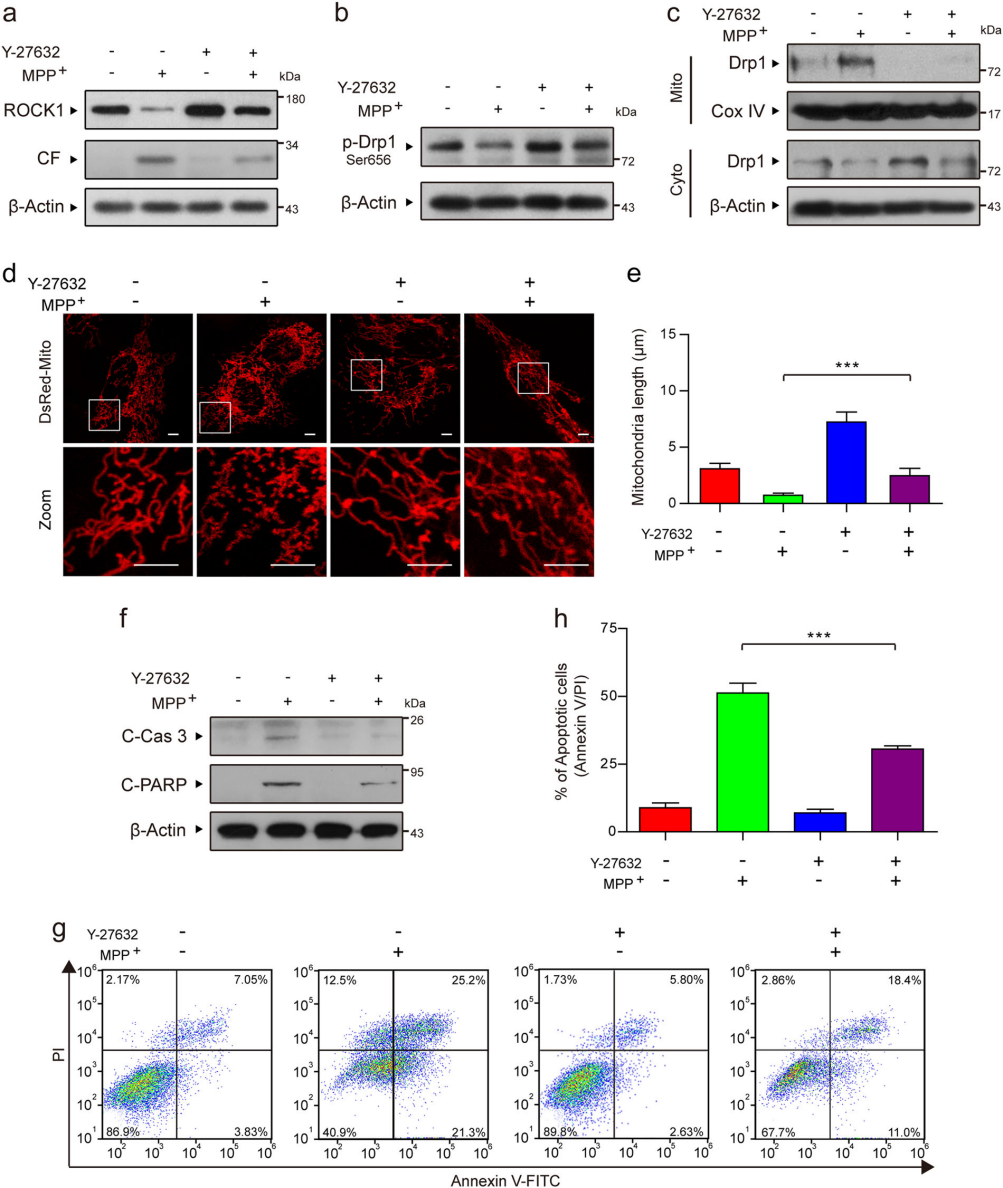
The ROCK1 activation inhibitor Y-27632 attenuates MPP^+^-induced Drp1-dependent aberrant mitochondrial fission and apoptosis through the inhibition of Drp1 dephosphorylation/activation. **a** PC12 cells were treated with MPP^+^ (1 mM) alone or pretreated with ROCK1 activation inhibitor Y-27632 (50 µM) for 2 h and then treated with MPP^+^ (1 mM) for 24 h. The expression of ROCK1 and CF of ROCK1 in whole-cell lysates was determined by western blot analysis. **b** The expression of p-Drp1 (Ser 656) was determined by western blot analysis. **c** The expression of Drp1 in mitochondrial lysates (Mito) and in cytosolic fractions (Cyto) was determined by western blot analysis. **d** Cells were transfected with a DsRed-Mito plasmid, and the mitochondria were viewed by confocal microscopy. Scale bars: 5 μm. **e** Mitochondrial length was quantified using the Imaris software. **f** The expression of C-Cas 3 and C-PARP in whole-cell lysates was determined by western blot analysis. **g**, **h** The apoptosis rate was measured by flow cytometry using annexin V-FITC/PI staining. The data are expressed as the means ± S.D. (*n* = 3). ***P < 0.001

To further verify the vital role of activated ROCK1 in MPP^+^-induced mitochondrial fission and apoptosis, we used Y-27632, a potent ROCK1 activation inhibitor. Preincubation of cells with Y-27632 before MPP^+^ treatment remarkably inhibited MPP^+^-induced ROCK1 activation, Drp1 dephosphorylation at Ser 656, and Drp1 mitochondrial translocation (Fig. 4a–c). Y-27632 also significantly blocked MPP^+^-mediated mitochondrial fission (Fig. 4d, e). Furthermore, Y-27632 markedly decreased the MPP^+^-induced activation of caspase-3 and PARP, as well as apoptosis (Fig. 4f–h). Collectively, our results confirm that activated ROCK1 plays a critical role in MPP^+^-induced Drp1-dependent mitochondrial fission and apoptosis in PD cell culture models.

### The ROCK1 activation inhibitor Y-27632 improves the symptoms of MPTP-induced PD mice by inhibiting Drp1-dependent aberrant mitochondrial fission and apoptosis

**Fig. 5:**
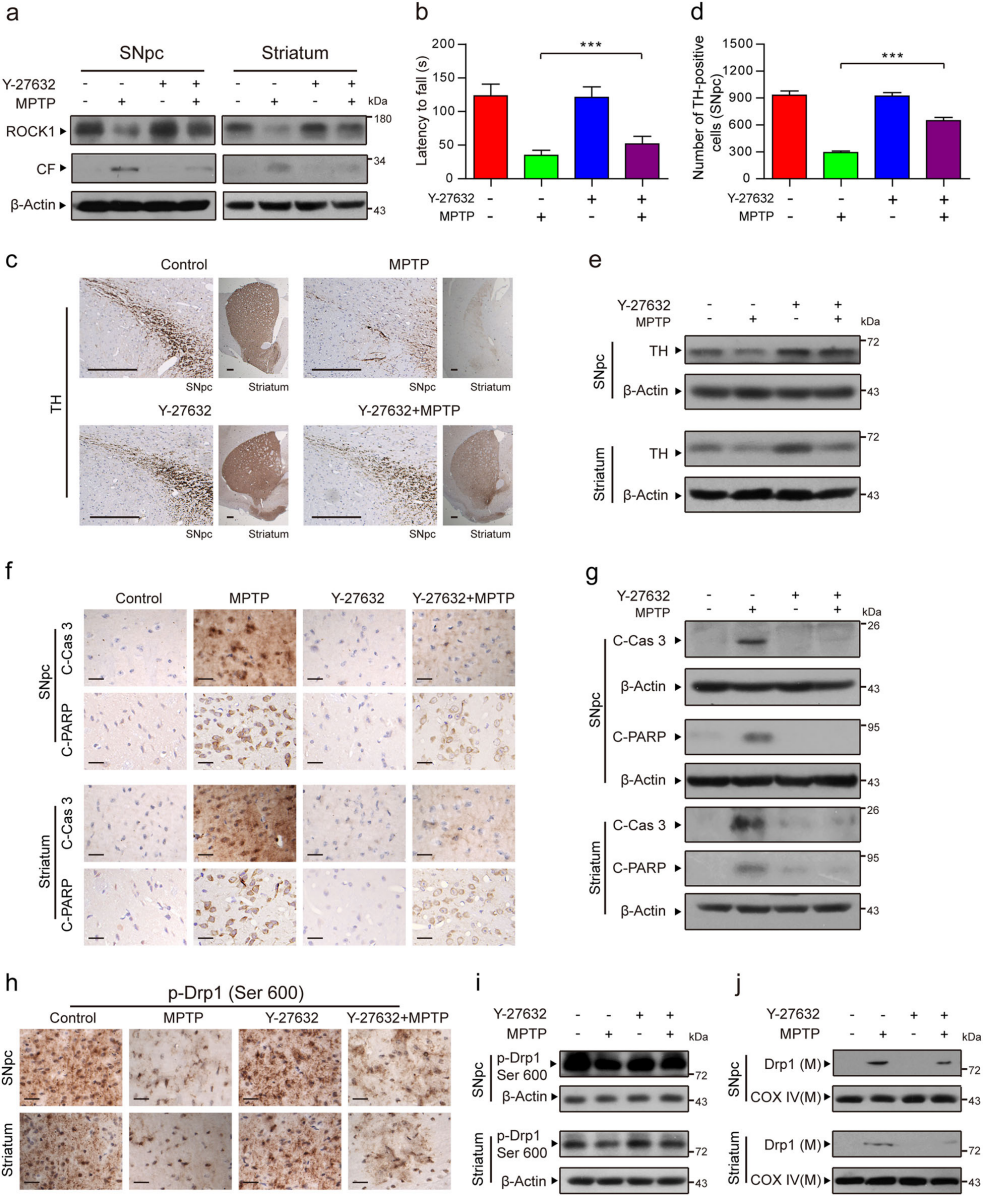
The ROCK1 activation inhibitor Y-27632 improves the symptoms of MPTP-induced PD mice by inhibiting Drp1-dependent aberrant mitochondrial fission and apoptosis. **a** The substantia nigra pars compacta (SNpc) of the midbrain and the striatum were prepared and used to detect the expression of ROCK1 and CF of ROCK1 using western blot analysis. **b** The latency for the mice to fall from the rotarod was recorded. **c** SNpc and striatal tissues from each group were fixed, dehydrated, and subjected to tyrosine hydroxylase (TH, a marker of dopaminergic nerve cells) staining for immunohistochemical analysis. Scale bars: 200 μm. **d** The number of TH-positive dopaminergic nerve cells in three randomly selected slices from each mouse was measured by Adobe Photoshop CC. **e** The expression of TH in the SNpc and striatum was determined by western blot analysis. **f** Immunohistochemical staining for C-Cas 3 and C-PARP in the SNpc and striatum are shown. Scale bars: 20 μm. **g** The expression of C-Cas 3 and C-PARP was determined by western blot analysis. **h** SNpc and striatal tissues from each group were subjected to p-Drp1 (Ser 600) staining for immunohistochemical analysis. Scale bars: 20 μm. **i** The expression of p-Drp1 (Ser 600) was also determined by western blot analysis. **j** The expression of Drp1 in mitochondrial lysates (M) was determined by western blot analysis. The data are expressed as the means ± S.D. (*n* = 3). ***P < 0.001

To verify whether our findings in vitro are consistent with those in vivo, MPTP (30 mg/kg/day, i.p.) was injected into C57BL/6 mice for 5 consecutive days to model PD. The mice in the Y-27632+MPTP group were injected with the specific ROCK1 inhibitor Y-27632 (5 mg/kg/day, i.p.) 30 min before MPTP treatment. Y-27632 remarkably inhibited the MPTP-induced cleavage/activation of ROCK1 both in the SNpc and the striatum of mice (Fig. 5a). As shown in Fig. 5b, the latency of the MPTP-treated PD mice to fall off the rotarod was significantly decreased, but pretreatment with Y-27632 before MPTP treatment rescued this decrease. Immunohistochemical analysis indicated that MPTP treatment remarkably decreased the number of TH-positive cells (tyrosine hydroxylase, TH, a marker of dopaminergic nerve cells), whereas Y-27632 reversed these changes (Fig. 5c, d). The results of TH expression, as detected by western blot analysis, were consistent with those of immunohistochemical staining (Fig. 5e). All of these findings suggest that our MPTP-induced PD mouse model was successfully established and that the inhibition of ROCK1 activation with Y-27632 can protect dopaminergic nerve cells from MPTP-mediated dopamine depletion in this in vivo model.

We next examined the mechanism underlying PD in vivo. Immunohistochemical and western blot analysis showed that injection with Y-27632 before MPTP treatment significantly inhibited the MPTP-mediated activation of caspase-3 and PARP (Fig. 5f, g). We also demonstrated that Y-27632 significantly decreased the MPTP-mediated dephosphorylation of Drp1 at Ser 600 in mice (corresponding to Ser 637 in humans, Fig. 3b) (Fig. 5h). Similarly, to further confirm that Y-27632 attenuates MPTP-induced Drp1 dephosphorylation at Ser 600 and subsequently the mitochondrial translocation of Drp1, the expression of phosphorylated Drp1 (Ser 600) and the expression of Drp1 in mitochondria were also detected by western blot analysis (Fig. 5i, j). Taken together, our findings indicate that an inhibitor of ROCK1 activation exerts neuroprotective effects in a PD mouse model by inhibiting Drp1-dependent aberrant mitochondrial fission and apoptosis, suggesting that ROCK1 and Drp1 may be potential therapeutic targets for PD.

## Discussion

In this work, we demonstrated that ROCK1 promotes dopaminergic nerve cell apoptosis by activating Drp1-mediated aberrant mitochondrial fission in vitro and in vivo. We also verified that the ROCK1 activation inhibitor Y-27632 has a therapeutic effect on a PD mouse model by suppressing Drp1-mediated aberrant mitochondrial fission and dopaminergic nerve cell apoptosis. Our study provides a mechanistic basis for the use of ROCK1 activation inhibitors for the treatment of PD.

Currently, neurotoxic models are broadly used as models of PD^38^. The dopaminergic neurotoxin MPTP (active metabolite: MPP^+^) originates from discoveries in the early 1980s and has been used extensively to generate animal models of PD^39,40^. MPTP contributes to the etiopathogenesis of PD by inducing mitochondria-targeted injury, decreasing dopamine levels and TH activity, and eliciting dopaminergic nerve cell apoptosis^12^. In the present study, we used MPP^+^-treated pheochromocytoma PC12 cells as an in vitro model of PD. We explored the molecular mechanisms underlying dopaminergic nerve cell apoptosis using MPP^+^ and MPTP-induced cell and animal models of PD, given their parallels with PD^41^. The morphology of the mitochondria changes from filamentous to punctate, with a marked decrease in mitochondrial length, due to mitochondrial fission^42–44^. Increasing evidence indicates that the mitochondrial protein Drp1 is required for mitochondrial fission and MPP^+^-induced neurotoxicity^12,32,35^. Moreover, mitochondria undergo fission to form punctate structures, inevitably leading to an increase in the number of mitochondria^45–48^. In this study, we demonstrated that the knockdown of Drp1 significantly inhibits MPP^+^-induced aberrant mitochondrial fission, decreases the number of mitochondria, and further blocks nerve cell apoptosis. Once Drp1 is activated, it is translocated to the outer mitochondrial membrane and forms a ring structure around the mitochondria, resulting in mitochondrial fission followed by Cyto C release and caspase activation, eventually leading to apoptosis^49^. In addition, Drp1 dephosphorylation at Ser 637 in humans has been shown to activate Drp1 and subsequently promote its translocation to the mitochondria and mitochondrial fission^19,32,50,51^. Consistent with these reports, our results also demonstrated that dephosphorylation of Drp1 at Ser 656/600 (Ser 656/600 in rats/mice corresponds to Ser 637 in humans) increases the mitochondrial translocation of Drp1 and leads to mitochondrial fission and apoptosis both in vitro and in vivo.

ROCK1 plays an important regulatory role in cell adhesion, migration, proliferation, and apoptosis^52^. ROCK1 is highly expressed in various cancer tissues^53^ and regulates apoptosis in various types of cancer cells^54^. Reportedly, ROCK1 can phosphorylate Drp1 at Ser 637 directly and lead to enhanced fission activity of Drp1^37^. Another report demonstrated that Drp1 is dephosphorylated at Ser 637 by calcineurin to cause its mitochondrial localization and promote mitochondrial fission^50,55^, and the inhibition of calcineurin activity blocks aberrant mitochondrial fission in PD^56^. Importantly, ROCK1 activation is required for calcineurin activation^57^. ROCK1 might indirectly dephosphorylate Drp1 through the induction of calcineurin activity. Whether ROCK1 directly phosphorylates Drp1 or indirectly dephosphorylates it through other phosphatases may be different in different cell lines. Our findings demonstrate that, in PD, ROCK1 is more likely to dephosphorylate Drp1 and induce mitochondrial fission during dopaminergic nerve cell apoptosis based on our evidence. First, the activation/cleavage of ROCK1 and the dephosphorylation/activation of Drp1 were found in our PD models. Second, the knockdown of ROCK1 remarkably decreased MPP^+^-induced dephosphorylation of Drp1, mitochondrial translocation of Drp1, aberrant mitochondrial fission, mitochondria number, and nerve cell apoptosis. Third, the ROCK1 activation inhibitor Y-27632 significantly improved symptoms in PD mice by inhibiting dephosphorylated Drp1, Drp1-mediated aberrant mitochondrial fission, and dopaminergic nerve cell apoptosis. In conclusion, our study reveals that ROCK1 plays a crucial role in regulating dopaminergic nerve cell apoptosis in PD via the dephosphorylation of Drp1 and the activation of Drp1-mediated aberrant mitochondrial fission.

It is well known that oxidative stress and microglia-mediated neuroinflammation are associated with the degeneration of neurons in MPTP-induced PD models. Increasing evidence has also shown that the Rho/ROCK signaling pathway plays a central role in inflammation and that ROCK inhibitors reportedly have neuroprotective effects^20,24^. In addition, it has been reported that Drp1 plays an important role in increasing the inflammatory response and reactive oxygen species production^58–62^. For example, ROCK1/Drp1-mediated dopaminergic neuronal death is believed to be related to mitochondrial fission through inflammation, and the ROCK1 activation inhibitor Y-27632 can exhibit neuroprotection via the inhibition of neuronal mitochondria fission.

In summary, the present findings indicate an important molecular mechanism of PD pathogenesis involving ROCK1-regulated dopaminergic nerve cell apoptosis. Importantly, for the first time, a mechanism was proposed by which ROCK1 cleavage/activation activates downstream Drp1 by dephosphorylating Drp1 and induces aberrant mitochondrial fission, eventually resulting in nigrostriatal dopaminergic nerve cell apoptosis and decreasing dopamine release. Collectively, our findings provide a better understanding of PD pathogenesis and a mechanistic basis for the use of ROCK1 activation inhibitors for the treatment of PD.

## Supplementary information

SUPPLEMENTAL MATERIAL

## References

[1] Zou YM, Liu J, Tian ZY, Lu D, Zhou YY (2015). Systematic review of the prevalence and incidence of Parkinson’s disease in the People’s Republic of China. Neuropsychiatr. Dis. Treat..

[2] Katzenschlager R, Lees AJ (2002). Treatment of Parkinson’s disease: levodopa as the first choice. J. Neurol..

[3] Cacabelos Ramón (2017). Parkinson’s Disease: From Pathogenesis to Pharmacogenomics. International Journal of Molecular Sciences.

[4] Giannoccaro MP, La Morgia C, Rizzo G, Carelli V, Mitochondrial DNA (2017). and primary mitochondrial dysfunction in Parkinson’s disease. Mov. Disord..

[5] Rappold PM (2014). Drp1 inhibition attenuates neurotoxicity and dopamine release deficits in vivo. Nat. Commun..

[6] Vives-Bauza C (2010). Control of mitochondrial integrity in Parkinson’s disease. Prog. Brain Res..

[7] Ishihara N, Otera H, Oka T, Mihara K (2013). Regulation and physiologic functions of GTPases in mitochondrial fusion and fission in mammals. Antioxid. Redox Signal..

[8] Inoue N (2018). Knockdown of the mitochondria-localized protein p13 protects against experimental parkinsonism. EMBO Rep..

[9] Wang WZ (2016). Parkinson’s disease-associated mutant VPS35 causes mitochondrial dysfunction by recycling DLP1 complexes. Nat. Med..

[10] Burte F, Carelli V, Chinnery PF, Yu-Wai-Man P (2015). Disturbed mitochondrial dynamics and neurodegenerative disorders. Nat. Rev. Neurol..

[11] Wang X (2012). LRRK2 regulates mitochondrial dynamics and function through direct interaction with DLP1. Hum. Mol. Genet..

[12] Wang XL (2011). DLP1-dependent mitochondrial fragmentation mediates 1-methyl-4-phenylpyridinium toxicity in neurons: implications for Parkinson’s disease. Aging Cell.

[13] Wang JX, Li Q, Li PF (2009). Apoptosis repressor with caspase recruitment domain contributes to chemotherapy resistance by abolishing mitochondrial fission mediated by dynamin-related protein-1. Cancer Res..

[14] Tanaka A, Youle RJ (2008). A chemical inhibitor of DRP1 uncouples mitochondrial fission and apoptosis. Mol. Cell.

[15] Estaquier J, Arnoult D (2007). Inhibiting Drp1-mediated mitochondrial fission selectively prevents the release of cytochrome c during apoptosis. Cell Death Differ..

[16] Frank S (2001). The role of dynamin-related protein 1, a mediator of mitochondrial fission, in apoptosis. Dev. Cell.

[17] Wei L, Surma M, Shi S, Lambert-Cheatham N, Shi J (2016). Novel insights into the roles of Rho kinase in cancer. Arch. Immunol. Ther. Exp. (Warsz.).

[18] Vemula S, Shi JJ, Hanneman P, Wei L, Kapur R (2010). ROCK1 functions as a suppressor of inflammatory cell migration by regulating PTEN phosphorylation and stability. Blood.

[19] Li GB (2015). Mitochondrial translocation and interaction of cofilin and Drp1 are required for erucin-induced mitochondrial fission and apoptosis. Oncotarget.

[20] He Q (2016). Inhibition of Rho-kinase by Fasudil protects dopamine neurons and attenuates inflammatory response in an intranasal lipopolysaccharide-mediated Parkinson’s model. Eur. J. Neurosci..

[21] Borrajo A, Rodriguez-Perez AI, Villar-Cheda B, Guerra MJ, Labandeira-Garcia JL (2014). Inhibition of the microglial response is essential for the neuroprotective effects of Rho-kinase inhibitors on MPTP-induced dopaminergic cell death. Neuropharmacology.

[22] Santos DB (2017). Succinobucol, a non-statin hypocholesterolemic drug, prevents premotor symptoms and nigrostriatal neurodegeneration in an experimental model of Parkinson’s disease. Mol. Neurobiol..

[23] Guo B (2017). Substantial protection against MPTP-associated Parkinson’s neurotoxicity in vitro and in vivo by anti-cancer agent SU4312 via activation of MEF2D and inhibition of MAO-B. Neuropharmacology.

[24] Villar-Cheda B (2012). Involvement of microglial RhoA/Rho-kinase pathway activation in the dopaminergic neuron death. Role of angiotensin via angiotensin type 1 receptors. Neurobiol. Dis..

[25] Singleterry J, Sreedhar A, Zhao YF (2014). Components of cancer metabolism and therapeutic interventions. Mitochondrion.

[26] Brandon M, Baldi P, Wallace DC (2006). Mitochondrial mutations in cancer. Oncogene.

[27] Skulachev VP (1999). Mitochondrial physiology and pathology; concepts of programmed death of organelles, cells and organisms. Mol. Asp. Med..

[28] Pokorny J (2014). Targeting mitochondria for cancer treatment - two types of mitochondrial dysfunction. Prague Med. Rep..

[29] Safe S (2015). Targeting apoptosis pathways in cancer–letter. Cancer Prev. Res. (Phila.).

[30] Murugan C (2016). Combinatorial nanocarrier based drug delivery approach for amalgamation of anti-tumor agents in breast cancer cells: an improved nanomedicine strategy. Sci. Rep..

[31] Adams JM, Cory S (2007). The Bcl-2 apoptotic switch in cancer development and therapy. Oncogene.

[32] Sheridan C, Martin SJ (2010). Mitochondrial fission/fusion dynamics and apoptosis. Mitochondrion.

[33] Perfettini JL, Roumier T, Kroemer G (2005). Mitochondrial fusion and fission in the control of apoptosis. Trends Cell Biol..

[34] Otera H, Mihara K (2011). Molecular mechanisms and physiologic functions of mitochondrial dynamics. J. Biochem..

[35] Knott AB, Perkins G, Schwarzenbacher R, Bossy-Wetzel E (2008). Mitochondrial fragmentation in neurodegeneration. Nat. Rev. Neurosci..

[36] Kang JH (2007). Phosphorylation of Rho-associated kinase (Rho-kinase/ROCK/ROK) substrates by protein kinases A and C. Biochimie.

[37] Wang WJ (2012). Mitochondrial fission triggered by hyperglycemia is mediated by ROCK1 activation in podocytes and endothelial cells. Cell Metab..

[38] Tieu K (2011). A guide to neurotoxic animal models of Parkinson’s disease. Cold Spring Harb. Perspect. Med..

[39] Davis GC (1979). Chronic Parkinsonism secondary to intravenous injection of meperidine analogues. Psychiatry Res..

[40] Langston JW, Ballard P, Tetrud JW, Irwin I (1983). Chronic Parkinsonism in humans due to a product of meperidine-analog synthesis. Science.

[41] Haque ME (2008). Cytoplasmic Pink1 activity protects neurons from dopaminergic neurotoxin MPTP. Proc. Natl Acad. Sci. USA.

[42] Bossy-Wetzel E, Barsoum MJ, Godzik A, Schwarzenbacher R, Lipton SA (2003). Mitochondrial fission in apoptosis, neurodegeneration and aging. Curr. Opin. Cell Biol..

[43] Karbowski M, Youle RJ (2003). Dynamics of mitochondrial morphology in healthy cells and during apoptosis. Cell Death Differ..

[44] Tondera D (2005). The mitochondrial protein MTP18 contributes to mitochondrial fission in mammalian cells. J. Cell Sci..

[45] Song W (2011). Mutant huntingtin binds the mitochondrial fission GTPase dynamin-related protein-1 and increases its enzymatic activity. Nat. Med..

[46] Rangaraju V, Lauterbach M, Schuman EM (2019). Spatially stable mitochondrial compartments fuel local translation during plasticity. Cell.

[47] Loson OC, Song Z, Chen H, Chan DC (2013). Fis1, Mff, MiD49, and MiD51 mediate Drp1 recruitment in mitochondrial fission. Mol. Biol. Cell.

[48] Kalia R (2018). Structural basis of mitochondrial receptor binding and constriction by DRP1. Nature.

[49] Chang CR, Blackstone C (2010). Dynamic regulation of mitochondrial fission through modification of the dynamin-related protein Drp1. Mitochondrial Res. Transl. Med..

[50] Cereghetti GM (2008). Dephosphorylation by calcineurin regulates translocation of Drp1 to mitochondria. Proc. Natl Acad. Sci. USA.

[51] Li GB (2017). Polyphyllin I induces mitophagic and apoptotic cell death in human breast cancer cells by increasing mitochondrial PINK1 levels. Oncotarget.

[52] Julian L, Olson MF (2014). Rho-associated coiled-coil containing kinases (ROCK): structure, regulation, and functions. Small GTPases.

[53] Lochhead PA, Wickman G, Mezna M, Olson MF (2010). Activating ROCK1 somatic mutations in human cancer. Oncogene.

[54] Tsai NP, Wei LN (2010). RhoA/ROCK1 signaling regulates stress granule formation and apoptosis. Cell Signal..

[55] Cribbs JT, Strack S (2007). Reversible phosphorylation of Drp1 by cyclic AMP-dependent protein kinase and calcineurin regulates mitochondrial fission and cell death. EMBO Rep..

[56] Buhlman L (2014). Functional interplay between Parkin and Drp1 in mitochondrial fission and clearance. Biochim. Biophys. Acta.

[57] Soudani N (2016). Calcineurin/NFAT activation-dependence of leptin synthesis and vascular growth in response to mechanical stretch. Front. Physiol..

[58] Santoro A (2017). DRP1 suppresses leptin and glucose sensing of POMC neurons. Cell Metab..

[59] Li A (2016). Metformin and resveratrol inhibit Drp1-mediated mitochondrial fission and prevent ER stress-associated NLRP3 inflammasome activation in the adipose tissue of diabetic mice. Mol. Cell. Endocrinol..

[60] Park J (2017). Anti-inflammatory effect of oleuropein on microglia through regulation of Drp1-dependent mitochondrial fission. J. Neuroimmunol..

[61] Zhou K (2017). RIP1-RIP3-DRP1 pathway regulates NLRP3 inflammasome activation following subarachnoid hemorrhage. Exp. Neurol..

[62] Zhang L (2017). Drp1-dependent mitochondrial fission mediates osteogenic dysfunction in inflammation through elevated production of reactive oxygen species. PLoS ONE.

