# Supplementary material for: ROCK1 induces dopaminergic nerve cell apoptosis via the activation of Drp1-mediated aberrant mitochondrial fission in Parkinson’s disease
Source: Exp Mol Med. 2019 Oct 2;51(10):116. doi: 10.1038/s12276-019-0318-z (PMC6802738; doi:10.1038/s12276-019-0318-z)
Supplement: Supplementary file 1 — SUPPLEMENTAL MATERIAL [file 12276_2019_318_MOESM1_ESM.doc]

Supplementary Material

**ROCK1 induces dopaminergic nerve cell apoptosis via activation of Drp1-mediated aberrant mitochondrial fission in Parkinson’s disease**

Qian Zhang et al.

***
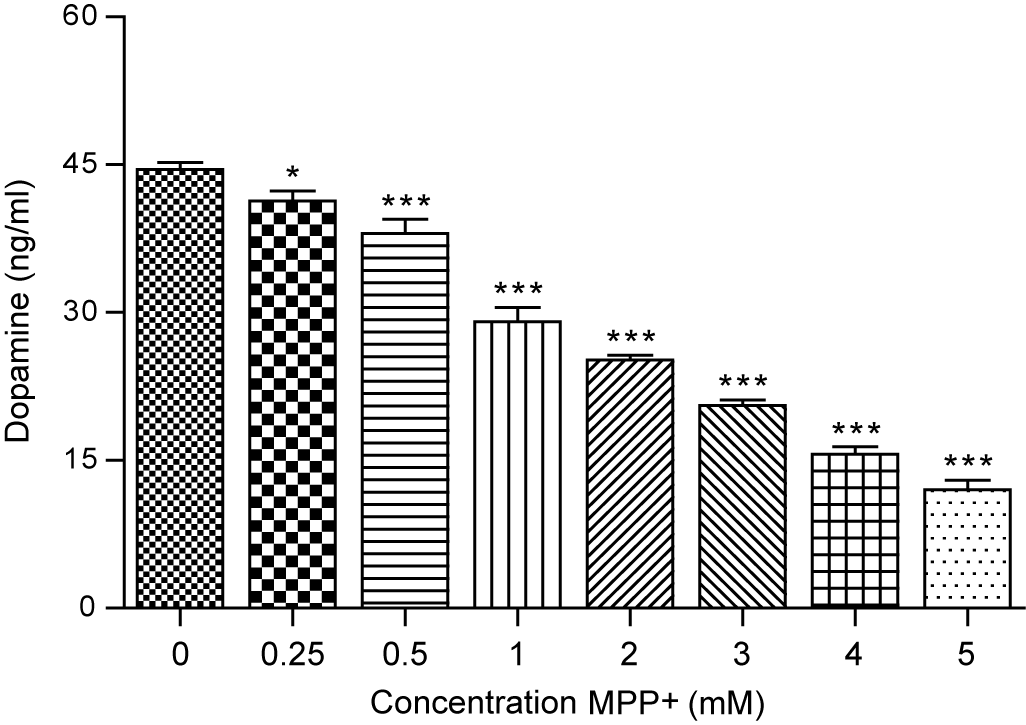
***

**Supplementary Fig. 1** MPP^+^ inhibits dopamine release in PC12 cells. PC12 cells were treated with MPP^+^ (0, 0.25, 0.5, 1, 2, 3, 4 and 5 mM) for 24 h. The release levels of dopamine were measured using ELISA. The data are expressed as the mean ± S.D. (n = 3). **P* < 0.05, ****P* < 0.001 vs. the control group.

**
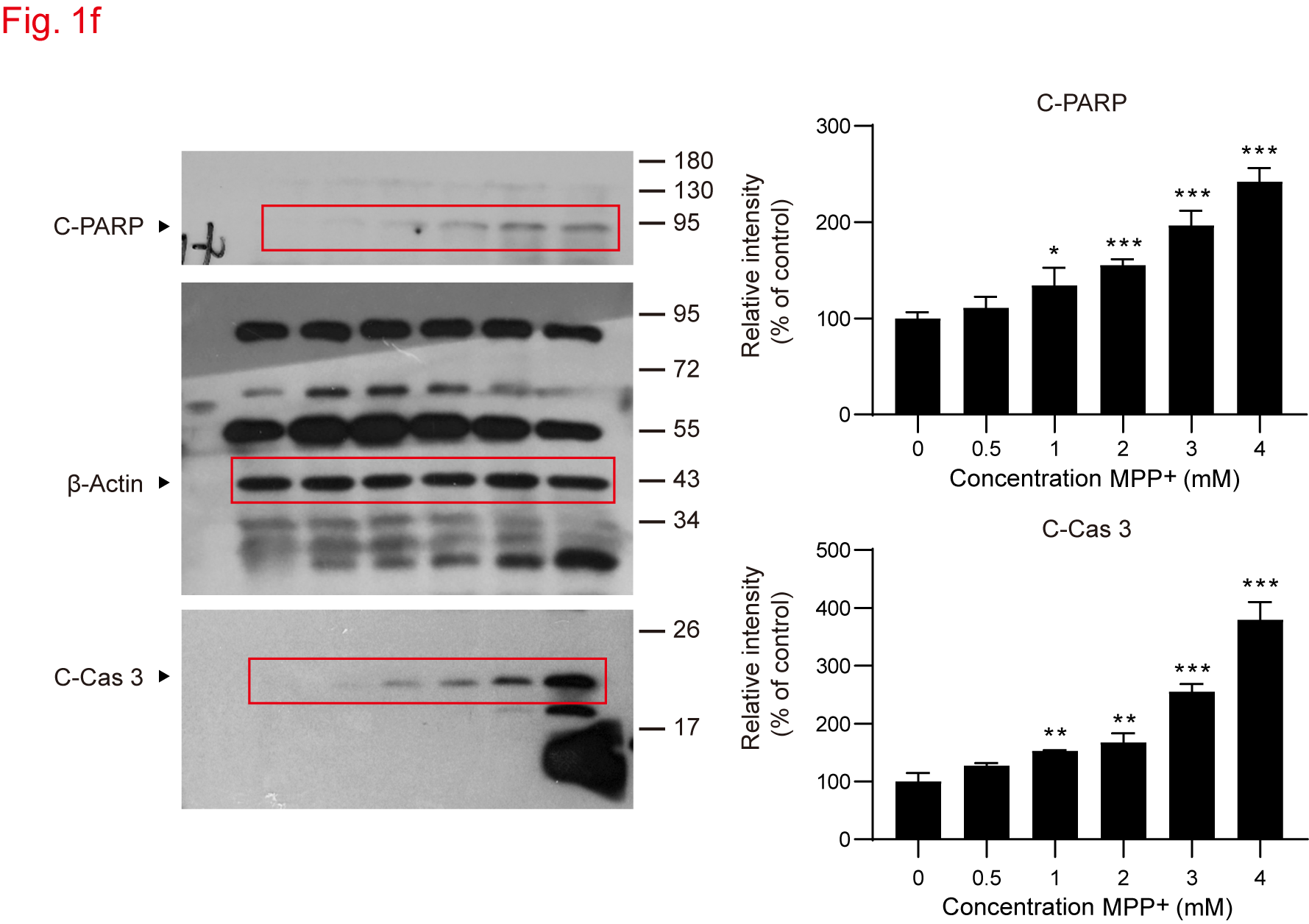
Supplementary Fig. 2** Original western blots images for Fig. 1f. Cropped areas are marked by red boxes. The data are expressed as the mean ± S.D. (n = 3). **P* < 0.05, ***P* < 0.01, ****P* < 0.001 vs. the control group.


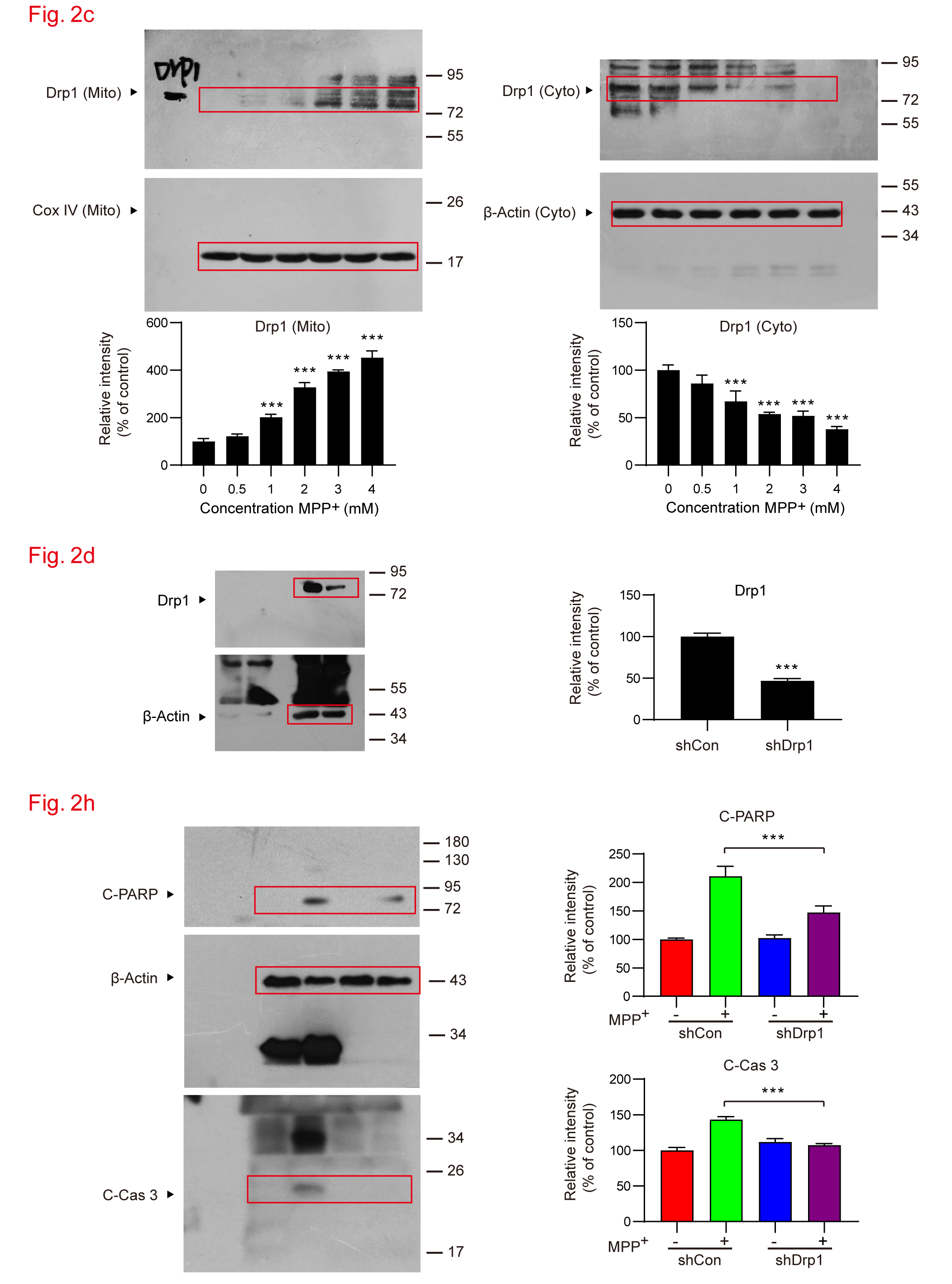


**Supplementary Fig. 3** Original western blots images for Fig. 2c, 2d and 2h. Cropped areas are marked by red boxes. Mito, mitochondrial lysates; Cyto, cytosolic fractions. The data are expressed as the mean ± S.D. (n = 3). **P* < 0.05, ****P* < 0.001 vs. the control group.

**
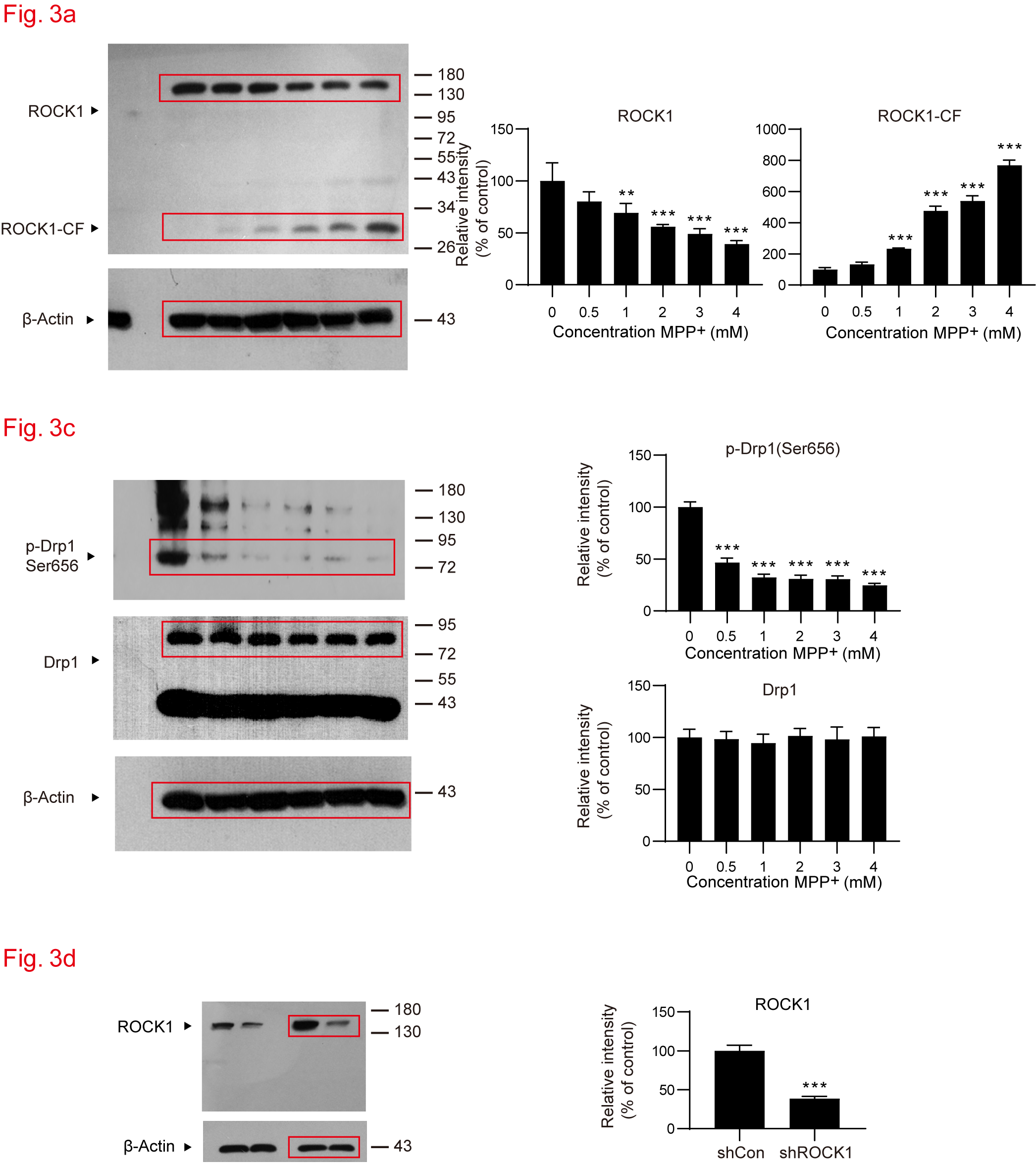
**

**Supplementary Fig. 4** Original western blots images for Fig. 3a, 3c and 3d. The data are expressed as the mean ± S.D. (n = 3). ***P* < 0.01, ****P* < 0.001 vs. the control group.


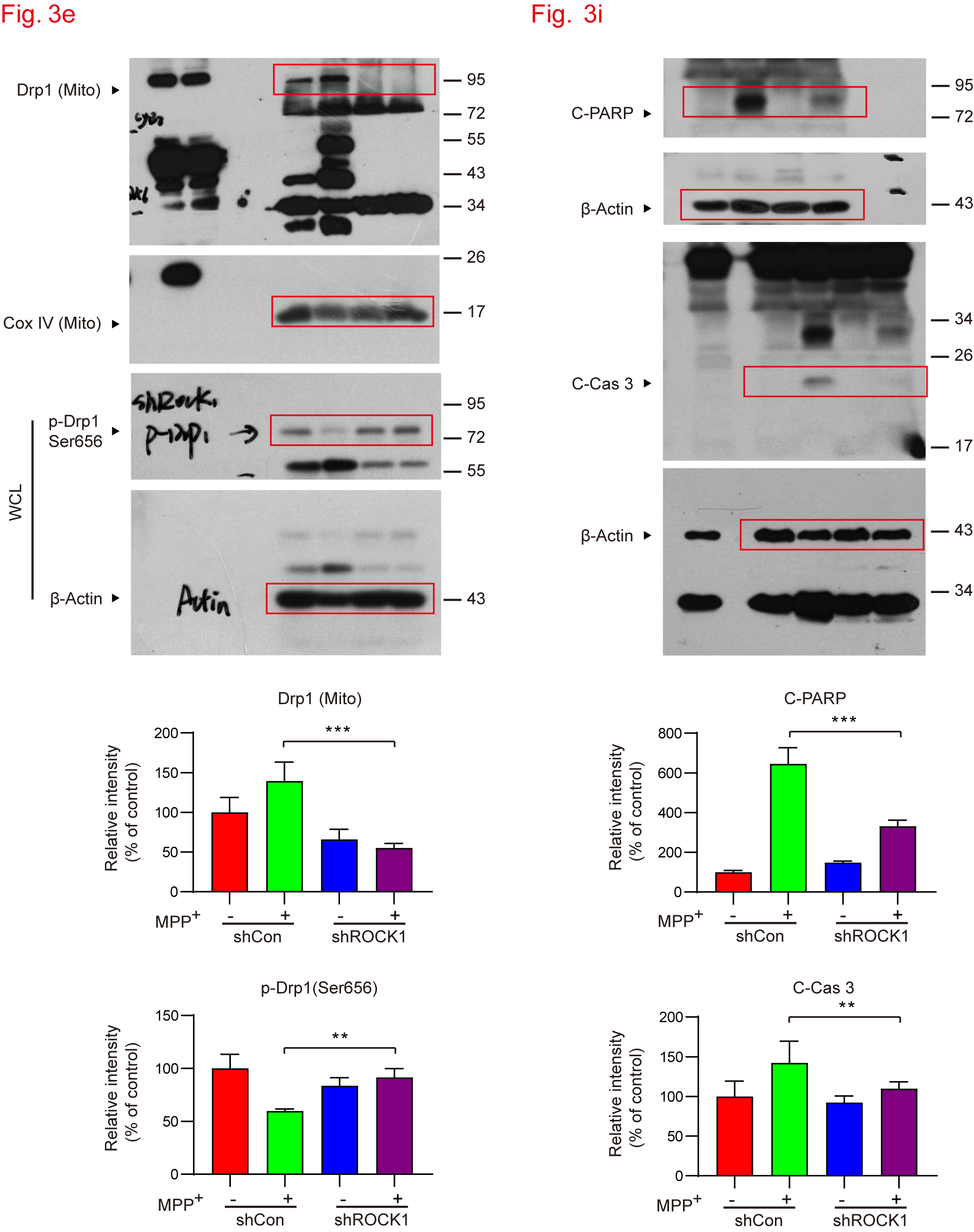


**Supplementary Fig. 5** Original western blots images for Fig. 3e and 3i. Cropped areas are marked by red boxes. WCL, whole-cell lysates; Mito, mitochondrial lysates. The data are expressed as the mean ± S.D. (n = 3). ***P* < 0.01, ****P* < 0.001 vs. the control group.

**
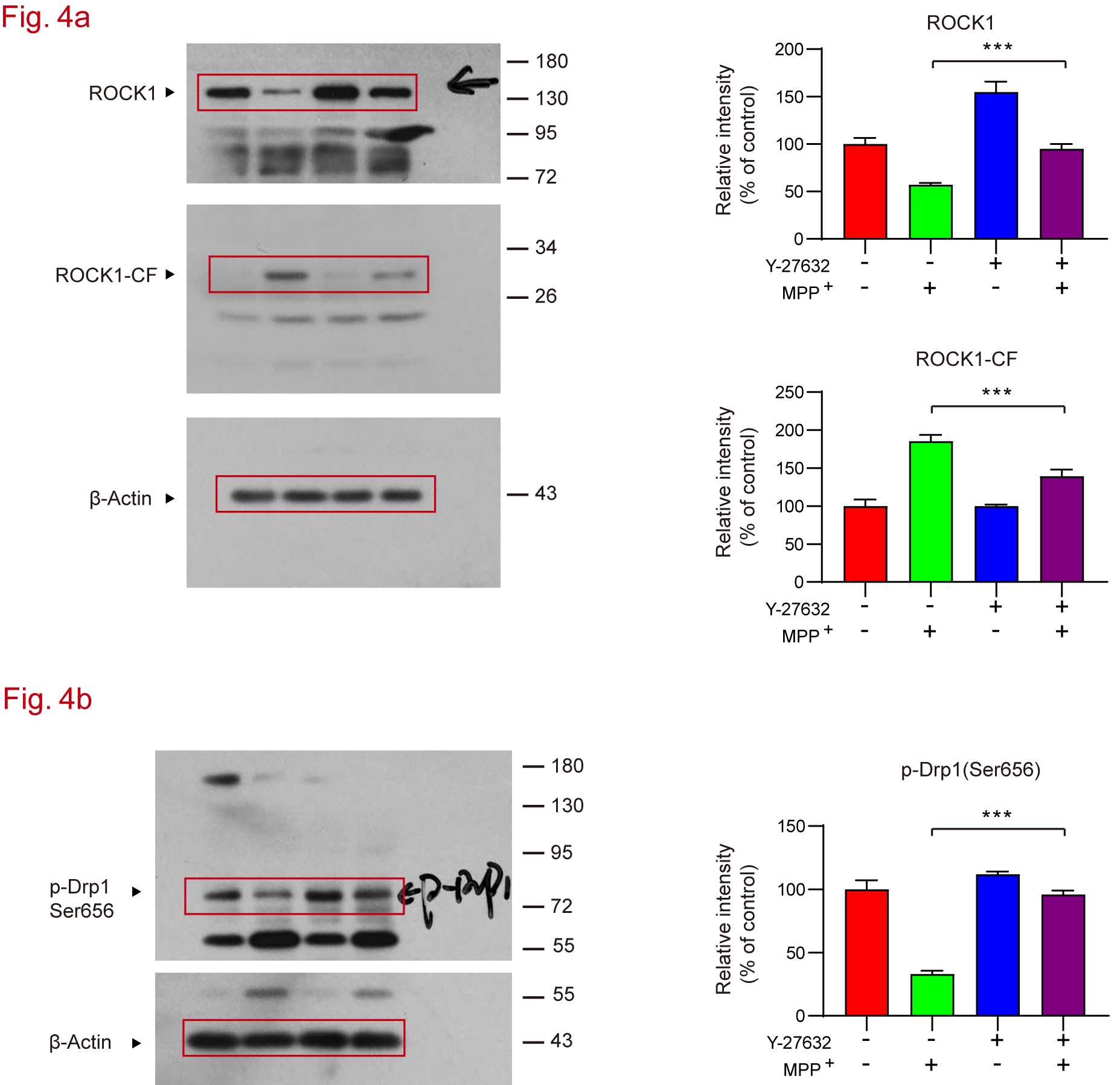
Supplementary Fig. 6** Original western blots images for Fig. 4a and 4b. Cropped areas are marked by red boxes. The data are expressed as the mean ± S.D. (n = 3). ***P* < 0.01, ****P* < 0.001 vs. the control group.


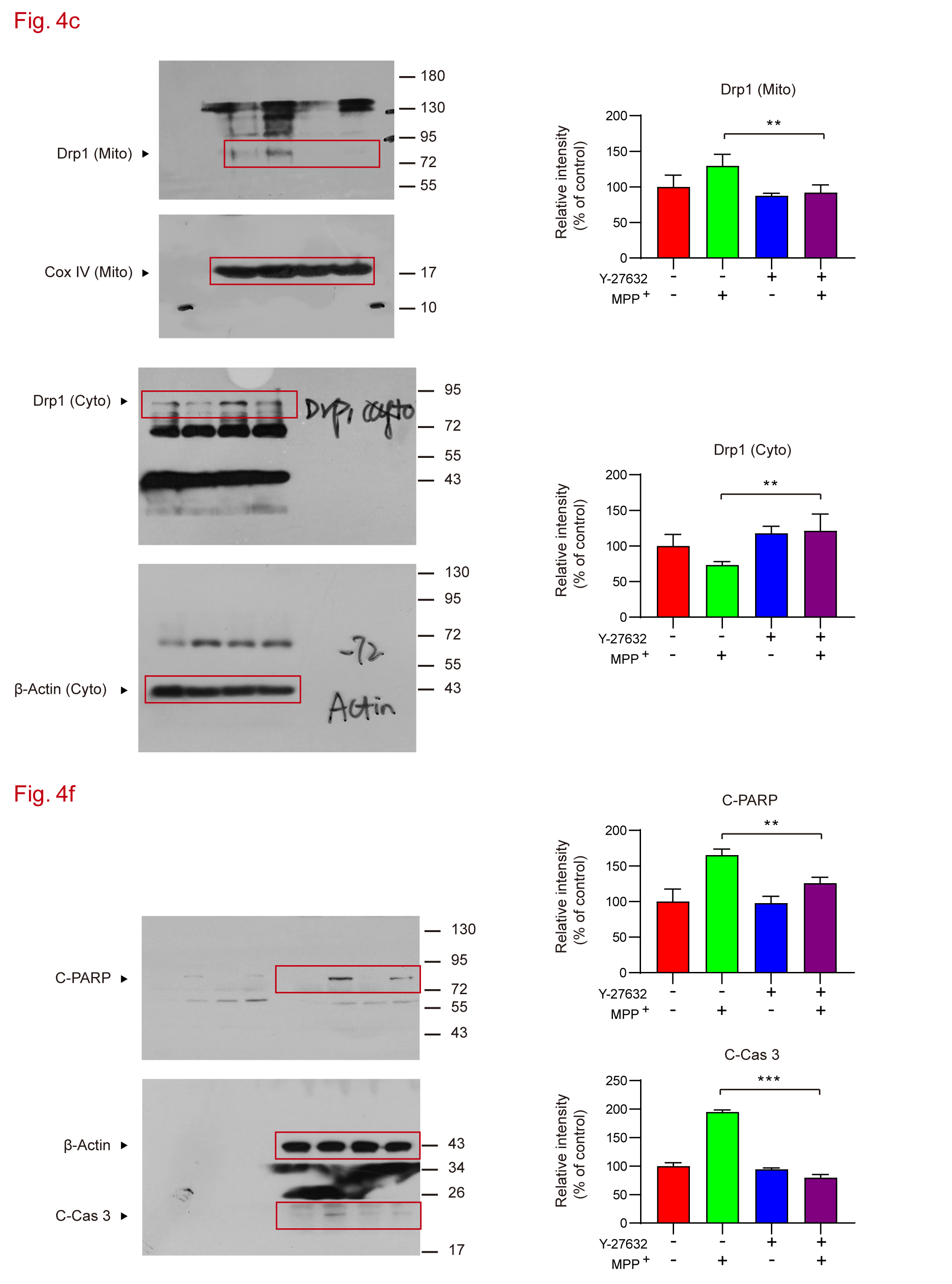


**Supplementary Fig. 7** Original western blots images for Fig. 4c and 4f. Cropped areas are marked by red boxes. Mito, mitochondrial lysates; Cyto, cytosolic fractions. The data are expressed as the mean ± S.D. (n = 3). ***P* < 0.01, ****P* < 0.001 vs. the control group.

**
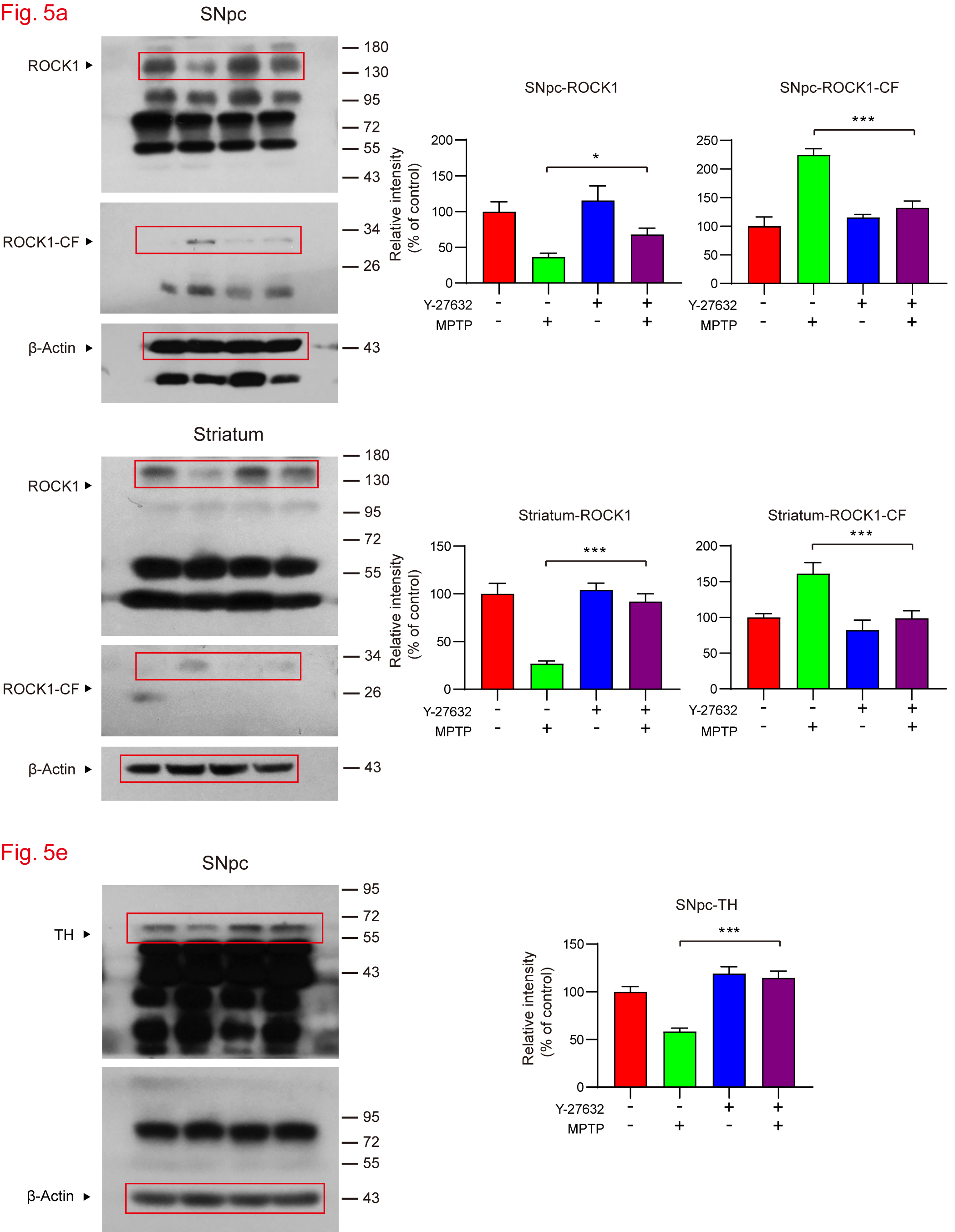
Supplementary Fig. 8** Original western blots images for Fig. 5a and 5e (SNpc-TH). Cropped areas are marked by red boxes. The data are expressed as the mean ± S.D. (n = 3). **P* < 0.05, ****P* < 0.001 vs. the control group.

**
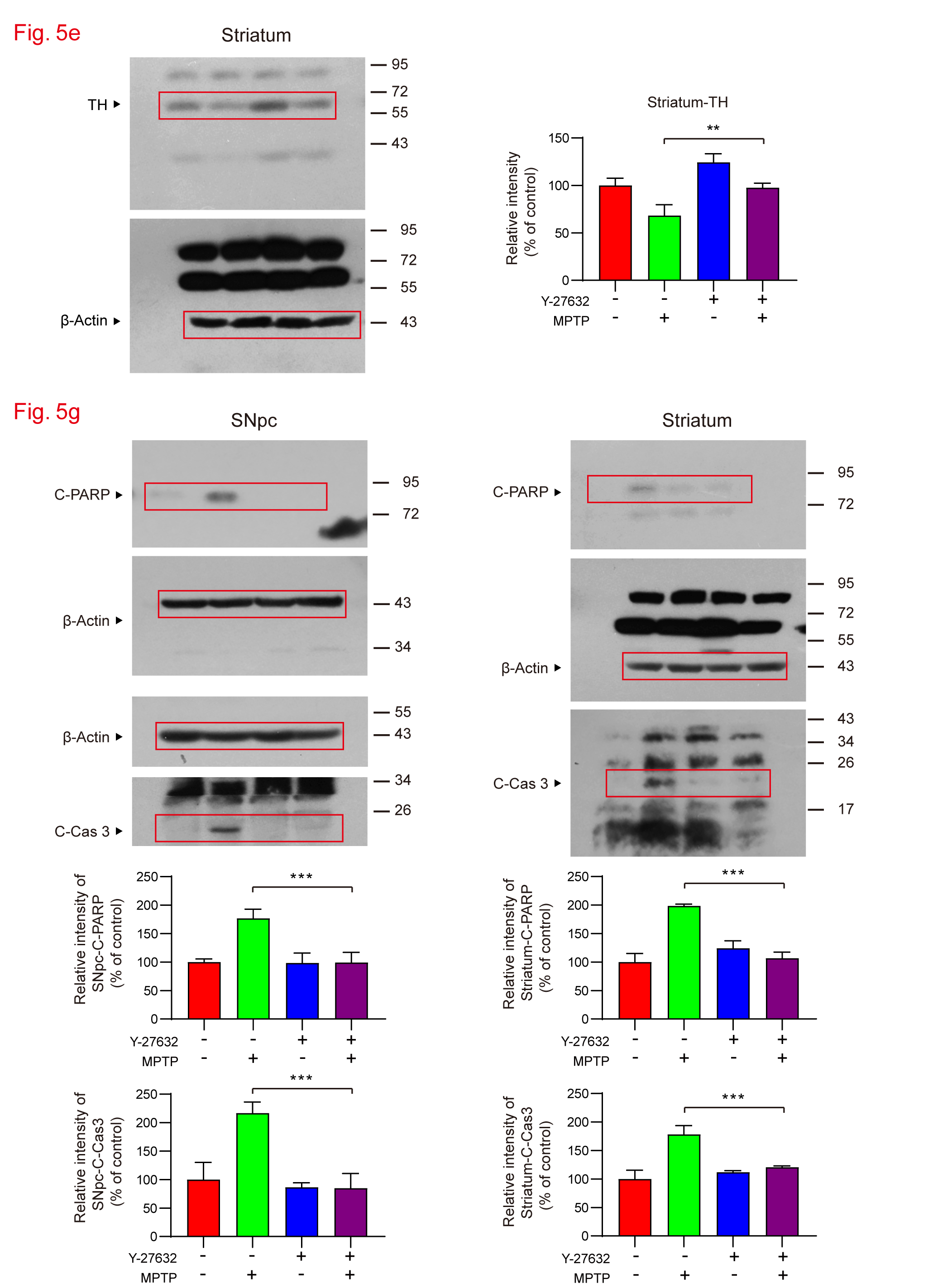
Supplementary Fig. 9** Original western blots images for Fig. 5e (Striatum-TH) and 5g. Cropped areas are marked by red boxes. The data are expressed as the mean ± S.D. (n = 3). ***P* < 0.01, ****P* < 0.001 vs. the control group.

**
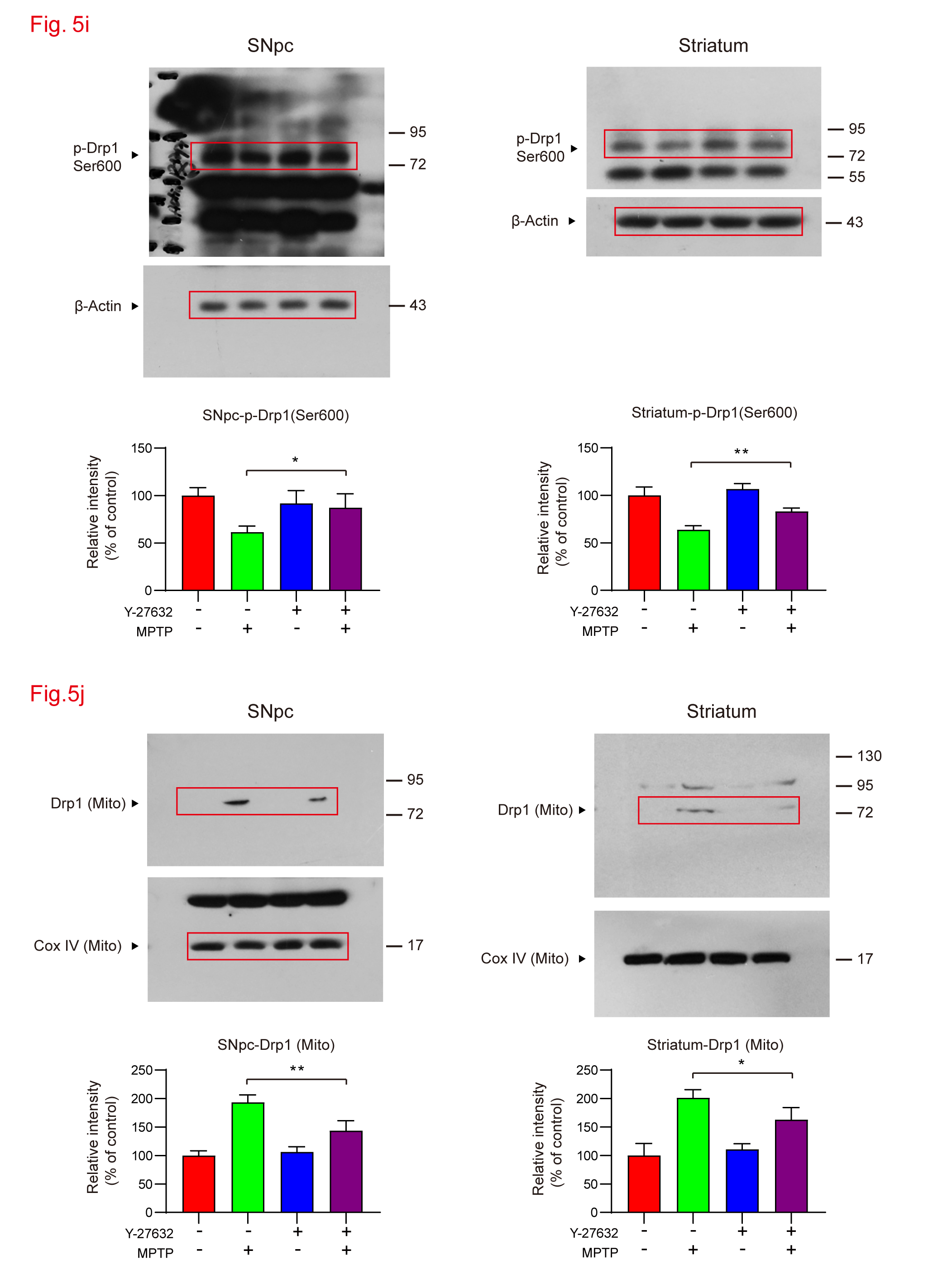
Supplementary Fig. 10** Original western blots images for 5i and 5j. Cropped areas are marked by red boxes. Mito, mitochondrial lysates. The data are expressed as the mean ± S.D. (n = 3). **P* < 0.05, ***P* < 0.01 vs. the control group.
